# Dissecting neural computations in the human auditory pathway using deep neural networks for speech

**DOI:** 10.1038/s41593-023-01468-4

**Published:** 2023-10-30

**Authors:** Yuanning Li, Gopala K. Anumanchipalli, Abdelrahman Mohamed, Peili Chen, Laurel H. Carney, Junfeng Lu, Jinsong Wu, Edward F. Chang

**Affiliations:** 1 Department of Neurological Surgery, University of California, San Francisco, San Francisco, CA USA; 2 Weill Institute for Neurosciences, University of California, San Francisco, San Francisco, CA USA; 3 Department of Electrical Engineering and Computer Science, University of California, Berkeley, Berkeley, CA USA; 4 Meta AI Research, Seattle, WA USA; 5 School of Biomedical Engineering & State Key Laboratory of Advanced Medical Materialsand Devices, ShanghaiTech University, Shanghai, China; 6 Department of Biomedical Engineering, University of Rochester, Rochester, NY USA; 7 Neurologic Surgery Department, Huashan Hospital, Shanghai Medical College, Fudan University, Shanghai, China; 8 Brain Function Laboratory, Neurosurgical Institute, Fudan University, Shanghai, China; 9 Present Address: School of Biomedical Engineering & State Key Laboratory of Advanced Medical Materials and Devices, ShanghaiTech University, Shanghai, China

**Keywords:** Neural encoding, Language, Cortex, Midbrain, Auditory system

## Abstract

The human auditory system extracts rich linguistic abstractions from speech signals. Traditional approaches to understanding this complex process have used linear feature-encoding models, with limited success. Artificial neural networks excel in speech recognition tasks and offer promising computational models of speech processing. We used speech representations in state-of-the-art deep neural network (DNN) models to investigate neural coding from the auditory nerve to the speech cortex. Representations in hierarchical layers of the DNN correlated well with the neural activity throughout the ascending auditory system. Unsupervised speech models performed at least as well as other purely supervised or fine-tuned models. Deeper DNN layers were better correlated with the neural activity in the higher-order auditory cortex, with computations aligned with phonemic and syllabic structures in speech. Accordingly, DNN models trained on either English or Mandarin predicted cortical responses in native speakers of each language. These results reveal convergence between DNN model representations and the biological auditory pathway, offering new approaches for modeling neural coding in the auditory cortex.

## Abstract (web summary)

Using direct intracranial recordings and modern speech AI models, Li and colleagues show representational and computational similarities between deep neural networks for self-supervised speech learning and the human auditory pathway.

## Main

Speech perception involves computations that transform acoustic signals into linguistic representations. Listening to speech activates the entire auditory pathway: from the auditory nerve (AN) and subcortical structures to the primary and nonprimary auditory cortical areas. Natural speech perception is a challenging task owing to variable acoustic cues for linguistic perceptual units (phonemes, syllables and words) under contextual factors such as interspeaker variability, emotional condition, prosody, coarticulation and speech rate^1–3^. Despite challenges, the auditory system is sensitive to this variability yet robustly extracts invariant phonetic and lexical information to support speech comprehension^2,4–6^. A central goal of speech and auditory neuroscience, as well as cognitive neuroscience in general, is to understand the computations performed by specific neural circuits and the representations generated by such computations^7^.

Classical cognitive models such as Cohort^8^, TRACE^9^ and their variants account for many psychological aspects of speech perception but do not explain neural coding or perform well in natural speech recognition. Conversely, classical neural encoding models^10–12^ explain neural coding during speech perception but cannot be directly adapted to a unified computational framework of speech perception. Modern artificial intelligence (AI) models using deep neural networks (DNNs) are approaching human-level performance in automatic speech recognition (ASR)^13–15^. However, their end-to-end ‘black box’ nature hampers the interpretation of internal computations and representations. Here, we aim to correlate DNN model computations and representations with the neural responses of the human auditory system to enhance the interpretability of AI models and offer new data-driven computational models of sensory perception.

Task-oriented pretrained DNN models have shown promise as computational models in sensory neuroscience. Using learned features from supervised learning tasks (for example, image recognition or sound classification), encoding models predict, with high accuracy, neural responses in the visual and auditory cortices^16–19^. In particular, Kell et al. used supervised convolutional neural networks (CNNs) to build encoding models for auditory responses in functional magnetic resonance imaging (fMRI) recordings and showed an aligned hierarchy between the CNNs and the auditory cortex^17^. Two of the key ingredients in DNN models are model architecture and training objective. Model architecture determines the computations performed on input signals, whereas the training objective affects representations learned through optimization. Neural coding in the ventral visual cortex is largely driven by spatial statistics in retinotopic space^20^, favoring CNNs with hierarchical spatial convolutions as computational models^16,19,21^.

Unlike core object recognition in vision modeling, which uses static images^22^, speech involves dynamic sequences often modeled by sequence-to-sequence (seq2seq) learning in modern AI^14,15,23^. These models extract dynamic representations of speech, shaped by both the current input (a nonlinear transformation of the current input) and the long-term dependencies in the input sequences (for example, the history of an input sequence). Furthermore, supervised model training, which often requires an enormous amount of labeled data, is not plausible as a generic learning strategy for the human auditory system. Human infants can learn phonetic and linguistic categories through speech sound statistics in native languages without explicit word learning^24,25^. Recent works have suggested unsupervised models without labeled data as models of vision and high-level language processing in the brain^26–28^. Therefore, unsupervised speech models capturing transient (local) and longer-context features of speech may yield more suitable speech perception models^29^.

This study directly compares state-of-the-art neural network models of speech to the human auditory pathway, aiming to uncover shared representations and computations between the two systems. Neural responses to natural speech across the ascending auditory pathway and the corresponding DNN speech embeddings are analyzed. Using a neural encoding framework^10,30^, we systematically evaluate the similarity between the auditory pathway and DNN models with different computational architectures (convolution, recurrence and self-attention) and training strategies (supervised and unsupervised objectives). Furthermore, inspection of DNN computations offers insights into the underlying mechanisms driving neural encoding predictions. Unlike previous modeling efforts that focused on a single language, mainly English, we here use a cross-linguistic paradigm to unveil language-invariant and language-specific aspects during speech perception.

In particular, we demonstrate the following findings: (1) the hierarchy in DNNs trained to learn speech representations correlates with that in the ascending auditory pathway; (2) unsupervised models without explicit linguistic knowledge can learn similar feature representations as the human auditory pathway; (3) deeper layers in speech DNNs correlate with speech-responsive populations in the nonprimary auditory cortex, driven by specific computations aligned with critical linguistically relevant temporal structures, such as phonemic and syllabic contexts; and (4) DNN-based models, unlike traditional linear encoding models, can reveal language-specific properties in cross-language speech perception. Taken together, our findings provide new data-driven approaches to modeling and evaluating neural coding in the auditory cortex.

## Results

### Overview

**Extended Data Fig. 1:**
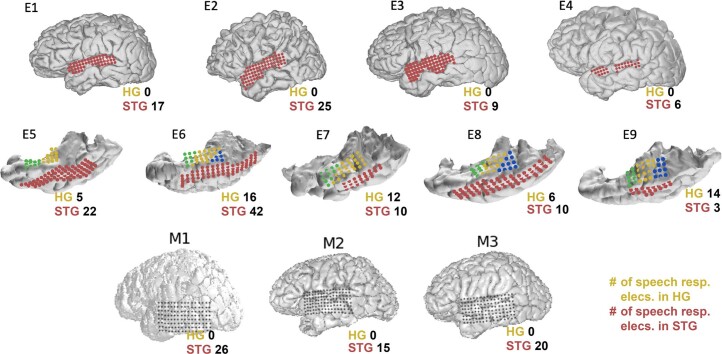
ECoG grid coverage for individual subjects. For E1-E9, electrodes are marked in colors according to anatomical label: superior temporal gyrus (red), Heschl’s gyrus (yellow), planum temporale (blue), planum polare (green). The numbers of significant speech responsive electrodes in each subject, sorted into anatomical areas, are summarized. (HG: Heschl’s Gyrus; STG: Superior Temporal Gyrus).

Our overall goal is to understand the computations and representations that occur and emerge throughout the auditory system during speech perception. To model the early pathway, we used a simulation of biophysical models of the auditory periphery and midbrain^31–33^, which have been highly successful at the cellular level. The biophysical model simulation yielded 50 distinct neurons in the AN and 100 distinct neurons in the inferior colliculus (IC). For the later portion of the pathway, we used intracranial cortical recordings from both the primary and nonprimary auditory cortical areas^34^ in nine participants (Extended Data Fig. 1). Local field potentials were recorded using high-density grids while these participants listened to English speech. A total of 553 electrodes were placed over the auditory cortex, 81 over the primary auditory cortex (Heschl gyrus (HG)) and 472 over the nonprimary auditory cortex (superior temporal gyrus (STG)). The amplitude of the local field potential in the high-gamma band (70–150 Hz) was used as a measure of local neuronal activity^35^. Neural responses across the early and late auditory systems were assessed using a set of 599 English sentences from the TIMIT corpus^36^.

We used five DNNs for the extraction of speech representations. These models differ in training objectives. In particular, we used two unsupervised models and three supervised models: (1) the HuBERT model, a transformer-based self-supervised model trained to predict masked portions of speech^15^; (2) the Wav2Vec 2 unsupervised model, a transformer-based self-supervised model trained for contrastive learning that distinguishes spans of a speech utterance from distractors^14^; (3) the Wav2Vec 2 supervised model, a transformer-based supervised model based on fine-tuning of the Wav2Vec 2 unsupervised model for ASR^14^; (4) the HuBERT/Wav2Vec 2 supervised model (HuBERT supervised), a fully supervised model trained only for supervised ASR and with no unsupervised pretraining; and (5) the Deep Speech 2 model, a long short-term memory (LSTM)-based supervised ASR model^13^. These models share a similar hierarchical framework: a multilayer convolutional feature encoder that extracts temporally constrained lower-level acoustic feature representations using one- and two-dimensional convolutions from a raw speech–audio waveform or spectrogram and a multilayer sequential encoder (with multiple transformer-encoder or recurrent (LSTM) layers) that extracts higher-level, context-dependent phonetic information from the CNN encoder output. We pretrained the speech-learning models on LibriSpeech, a standard corpus of 960 h of continuous naturalistic English speech^37^ (Table 1).

**Table 1:** Summary of network training objectives and architectures

Models	Unsupervised objective	Supervised objective	Architecture	ASR task performance (word error rate (%))
HuBERT^15^	Masked prediction	NA	7 CNN layers + 12 transformer-encoder layers	6 (after fine-tuning)
Wav2Vec 2 (unsupervised)^14^	Contrastive learning	NA	7 CNN layers + 12 transformer-encoder layers	6.3 (after fine-tuning)
Wav2Vec 2 (supervised)^14^	Contrastive learning	ASR	7 CNN layers + 12 transformer-encoder layers	6.3
HuBERT/Wav2Vec 2 (pure supervised)	NA	ASR	7 CNN layers + 12 transformer-encoder layers	7.4
Deep Speech 2^13^	NA	ASR	3 CNN layers + 5 LSTM layers	8.00

**Fig. 1:**
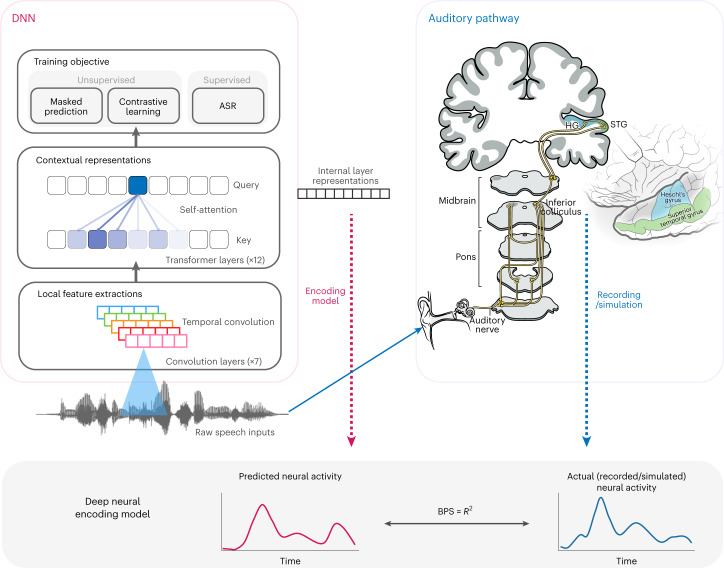
Overall framework for comparing representations in DNNs and the auditory pathway. The architecture of a family of DNN models, HuBERT/Wav2Vec 2, is illustrated on the left. The auditory pathway is illustrated on the right, with highlighted areas indicating the locations of the recorded/simulated electrophysiology signals. The same natural speech stimuli were presented to both the human participants and the DNN models, and the internal activations of each DNN layer were extracted and aligned with the corresponding neural activity from each recording site in the auditory pathway. A ridge regression model was fitted to predict neural activity from time-windowed DNN representations, and the regression coefficient of determination *R*^2^ between the predicted and actual neural activity was used as a metric of prediction accuracy.

The speech responses from the auditory pathway and DNNs were aligned in time to train linear encoding models. Different representation layers in the DNNs were used to predict neural responses in the auditory pathway (Fig. 1). The performance of these models (prediction *R*^2^) quantifies the similarity between the DNN-learned speech representations and the underlying neural representations. In this way, we tested the hypothesis that speech DNN models converge to a similar representation hierarchy as the ascending auditory pathway. NA, not applicable.

**Extended Data Fig. 2:**
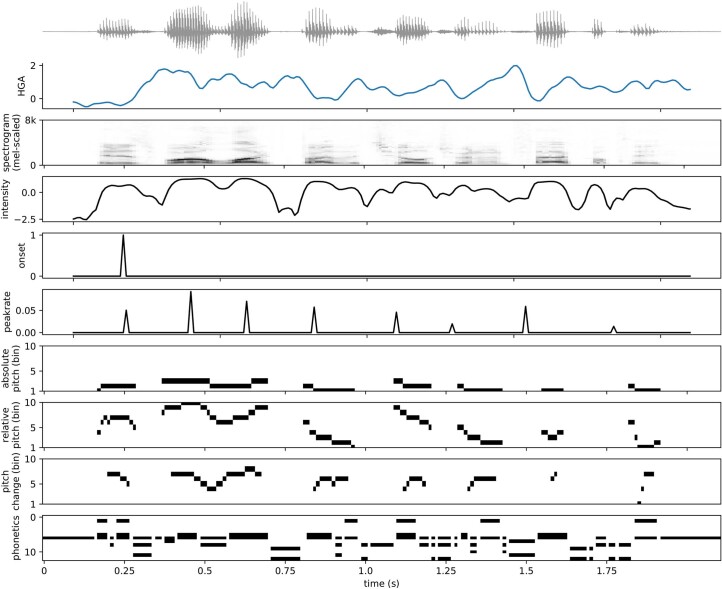
Acoustic phonetic feature encoding model. Example of feature extraction for a sample sentence, read by a male speaker:’It is well liked by the children and faculty.’ From top to bottom: 1) raw waveform; 2) high-gamma (z-scored) activity at an example electrode; 3) Mel-scaled spectrogram; 4) intensity of voicing; 5) sentence onset; 6) time course of peak rate; 7) absolute pitch (binned into 10 bins); 8) relative pitch (binned into 10 bins); 9) pitch change (binned into 10 bins); 10) phonetic features.

To address heterogeneous signal-to-noise ratios across the auditory pathway areas, participants and signal modalities, we established benchmark baselines for each electrode and neuron. For each recording site, we trained two baseline models: (1) a linear temporal receptive field (TRF) model using spectrogram features^10^ and (2) a linear TRF model using acoustic–phonetic features, including spectrogram, speech envelope/temporal landmark, pitch and phonetic features^34^ (Extended Data Fig. 2). The performance of neural encoding models using different sets of features was normalized against the second baseline model with a heuristic full-feature set in each recording site to make evaluations comparable across sites and areas. This normalized prediction *R*^2^ was termed the brain-prediction score (BPS), a primary metric for prediction accuracy at each site.

### DNN hierarchy correlates with the ascending auditory pathway

We tested whether DNNs trained to learn speech representations converge on the same standard auditory (serial feedforward) hierarchy of AN–IC–HG–STG. To do this, we compared the DNN hierarchy and the ascending auditory pathway from two different perspectives: (1) does the hierarchy of layers in DNNs mirror a similar hierarchy in the ascending auditory pathway? (2) Are the feature representations learned by DNNs more strongly correlated with neural coding than linguistically derived acoustic–phonetic feature sets?

**Fig. 2:**
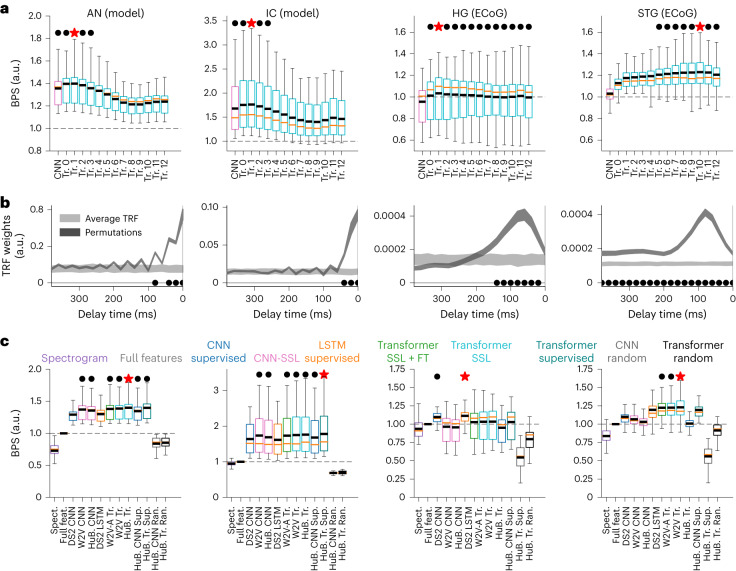
Hierarchy of layers in DNNs correlates with the AN–midbrain–STG ascending auditory pathway. **a**, Normalized BPS of the best-performing neural encoding model based on every single layer in the HuBERT model (maximum over delay window lengths). Magenta bars indicate CNN output layers; cyan bars indicate transformer layers. Red star indicates the best model for each area; black dot indicates other models that were not statistically different from the best model (*P* > 0.05, two-sided paired *t* test; *n* = 50 neurons for the AN, *n* = 100 neurons for the IC, *n* = 53 electrodes for the HG, *n* = 144 electrodes for the STG). From left to right: AN, IC, HG and STG (same for each row in **b** and **c**). **b**, Averaged TRF weights (absolute beta weights of the spectrotemporal encoding model) in speech-responsive units/electrodes of each area (mean ± s.e.m.; light-shaded areas indicate random permuted distributions; black dots indicate time points with TRF weights significantly higher than the chance level; *t* test, two-sided *P* < 0.05, Bonferroni-corrected for 20 time points). **c**, Normalized BPS of the best-performing neural encoding model (maximum over single layers and delay window lengths) for different areas of the pathway. Color key indicates different layer types (CNN supervised, CNN layers from the supervised Deep Speech 2 model or HuBERT supervised model; CNN-SSL, CNN layers from the self-supervised Wav2Vec 2 or HuBERT model; LSTM supervised, LSTM layers from Deep Speech 2; Transformer SSL + FT, transformer layers from the self-supervised and fine-tuned Wav2Vec 2 model; Transformer SSL, transformer layers from the self-supervised Wav2Vec 2 or HuBERT model; Transformer supervised, transformer layers from the pure supervised HuBERT model; CNN random, CNN layers from the randomized HuBERT model; Transformer random, transformer layers from the randomized HuBERT model). Red star indicates the best model for each area; black dot indicates other models that were not statistically different from the best model (*P* > 0.05, two-sided paired *t* test). Dashed horizontal line indicates the baseline model using full acoustic–phonetic features. For **a** and **c**, the box plot shows the first and third quantiles across electrodes (orange line indicates the median; black line indicates the mean value; whiskers indicate the 5th and 95th percentiles). a.u., arbitrary units; ECoG, electrocorticography; Spect, spectrogram; feat., features; DS2, Deep Speech 2; W2V, Wav2Vec 2; HuB., HuBERT; W2V-A, Wav2Vec 2 ASR supervised model; Tr., transformer; Sup., supervised; Ran., randomized. Source data

**Extended Data Fig. 3:**
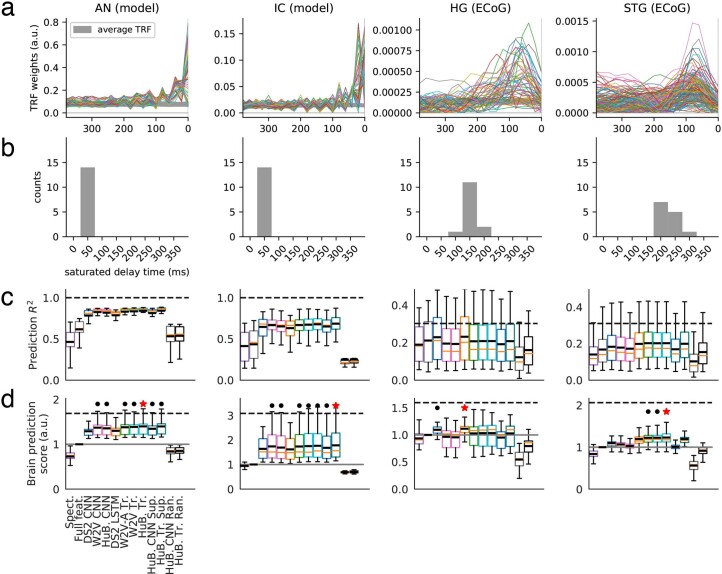
Temporal profile, raw encoding *R*^2^, and noise ceiling. **a)** The temporal receptive field (absolute beta weights of the spectrotemporal encoding model) of each individual speech-responsive unit/electrode. (Gray shaded areas indicate random permuted distributions of the averaged TRF across all units/electrodes, same as Fig. 2c. **b)** The histogram of the optimal delay window lengths corresponding to models in Fig. 2a. **c)** raw prediction R2 of different models. Dashed line: noise ceiling estimated from 10 repeated trials. **d)** Distribution of the normalized brain prediction score of each model across individual units/electrodes. Dashed line: noise ceiling estimated from 10 repeated trials. Red star (*) indicates the best model for each area, black dot (.) indicates other models that are not statistically different from the best model (p > 0.05, two-sided paired t-test; n = 50 neurons for AN; n = 100 neurons for IC; n = 53 electrodes for HG; n = 144 electrodes for STG). Box plot shows the first and third quantiles across electrodes, orange line indicates the median, black line is the mean value, and whiskers indicate the 5^th^ and 95^th^ percentiles. Source data

**Extended Data Fig. 4:**
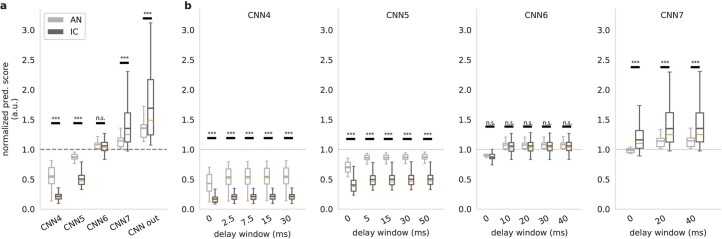
Comparing DNN encoding performance across different convolutional layers in the HuBERT model for AN and IC neurons. **a)** The brain prediction score of the best-performing neural encoding model based on each single layer (the 4^th^ – 7^th^ CNN layers and the final convolution output) in the HuBERT model model (maximum over delay window length). **b)** The averaged brain prediction score at CNN4 – CNN7 in the HuBERT model with different delay window lengths. Note that the sampling rates vary at different layers: CNN4–400 Hz, CNN5 – 200 Hz, CNN6 – 100 Hz, CNN7 & CNN out – 50 Hz. AN: light shaded bars; IC: dark shaded bars. Box plot shows the first and third quantiles across electrodes, orange line indicates the median, gray line is the mean value, and whiskers indicate the 5^th^ and 95^th^ percentiles. * *p* < 0.05, ** *p* < 0.01, *** *p* < 0.001, two-sample t-test, two-sided, n = 50 unique neurons for AN, n = 100 unique neurons for IC. Source data

**Extended Data Fig. 5:**
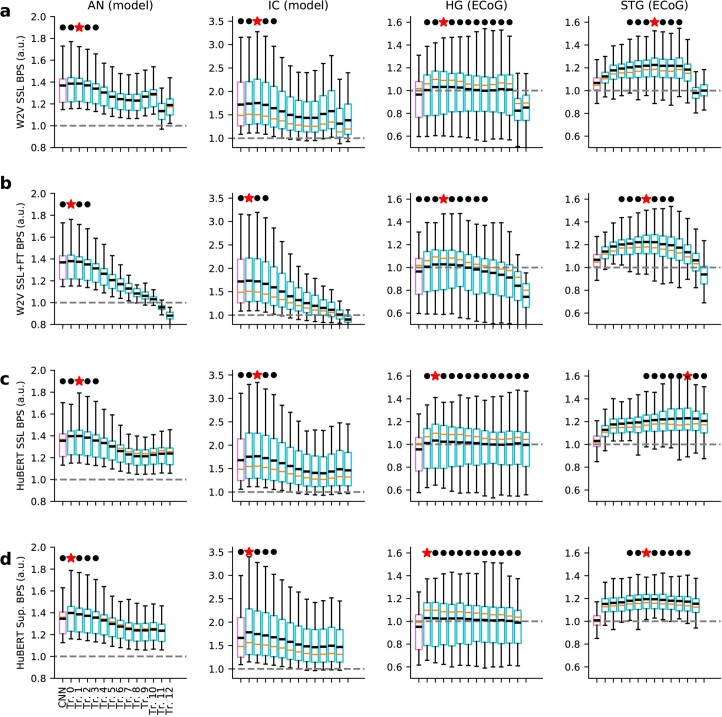
Comparing DNN encoding performance across different models. The distribution of the normalized brain prediction score of the best-performing neural encoding model based on each single layer in the DNN model (maximum over delay window length) across individual electrodes. **a**) Wav2Vec 2.0 Unsupervised (SSL) model; **b**) Wav2Vec 2.0 Supervised finetuning (SSL + FT) model; **c**) HuBERT Unsupervised (SSL) model; **d)** HuBERT pure supervised model. Each column corresponds to one area in the auditory pathway, from left to right AN/IC/HG/STG. Magenta bars indicate CNN output layers, cyan bars indicate Transformer layers. Red star (*) indicates the best model for each area, black dot (.) indicates other models that are not statistically different from the best model (p > 0.05, two-sided paired t-test). Box plot shows the first and third quantiles across electrodes, orange line indicates the median, black line is the mean value, and whiskers indicate the 5^th^ and 95^th^ percentiles. Source data

First, we considered a representative state-of-the-art self-supervised DNN, the HuBERT model^15^. For every single-layer representation model in HuBERT, we computed the averaged BPS (normalized prediction *R*^2^) across all recording sites within each anatomical area (Fig. 2; see Extended Data Fig. 3 for raw *R*^2^ and noise-ceiling values). Compared to the linear model with heuristic acoustic–phonetic features, the performance of the DNN encoding model was 39.9% higher in the AN at transformer layer 1 (mean BPS = 1.399, *t*(50) = 13.97, *P* = 2.5 × 10^−44^, two-sided), 76.3% higher in the IC at transformer layer 1 (mean BPS = 1.763, *t*(100) = 13.75, *P* = 5 × 10^−43^, two-sided), 3.4% higher in the HG at transformer layer 1 (mean BPS = 1.033, *t*(53) = 1.20, *P* = 0.23, two-sided) and 23.0% higher in the STG at transformer layer 10 (mean BPS = 1.230, *t*(144) = 16.1, *P* = 5 × 10^−^^58^) (Fig. 2a). Moreover, of all layers in the same unsupervised DNN model, the CNN layers and the first four transformer layers in the hierarchy best predicted the AN and IC responses (Fig. 2a). A finer-grain analysis suggested that the early part of the CNN layers predicted AN responses better than IC responses, whereas the late part of the CNN layers predicted IC responses better than AN responses (Extended Data Fig. 4). The activity of the speech-responsive STG population was best predicted by the later part of the DNN model and peaked at the tenth layer out of all 12 transformer layers (Fig. 2a). HG responses were predicted equally well by all transformer layers. However, none of these layers of speech DNNs outperformed the baseline acoustic model in predicting HG responses (Fig. 2a). Furthermore, this general hierarchical trend was consistent across several DNN models that shared a similar architecture with the HuBERT model but with different training objectives (Extended Data Fig. 5).

Next, we tested the hypothesis that the auditory hierarchy is characterized by increasingly long windows of temporal integration. Using the baseline spectrogram model, we found that the TRFs estimated for each area showed a hierarchy of progressive temporal integration of acoustic inputs: temporal responses in the peripheral areas AN and IC were mostly transient within 100 ms, whereas neural responses in the cortex showed integration time windows longer than 100 ms. More specifically, HG responses on average had a consistent temporal integration window of 200 ms, and some STG electrodes showed a significant sustained temporal integration window of up to 300 ms and longer (Fig. 2b and Extended Data Fig. 3). This trend of increasing temporal integration window was also consistent with the estimated optimal encoding window size that yielded the best prediction in encoding models (Extended Data Fig. 3).

Finally, we generalized the evaluations to a set of different DNN models (Table 1). We found that, for all areas, all DNN-based encoding models outperformed the baseline linear models. On average, compared to the linear model using heuristic acoustic–phonetic features, DNN-based encoding models explained 29.3–40.0% more variance in the AN, 61.7–76.3% more variance in the IC, −3.5% to 11.4% more variance in the HG and 3.1–23.0% more variance in the STG (Fig. 2c). In particular, the transformer layers in the unsupervised HuBERT model achieved the highest average performance in all areas except the HG. Moreover, we found that neural responses to speech in the auditory periphery (AN and IC) and primary auditory cortex (HG) were also largely characterized by locally resolved filters such as CNN representations, which had a fixed finite receptive field in time (*P* > 0.05 compared to HuBERT, two-sided *t* test; Fig. 2c). In contrast, speech responses in the nonprimary auditory cortex (STG) were better predicted using the deeper transformer layers in the DNNs (Fig. 2c and Extended Data Figs. 4 and 5).

To sum up from the above three perspectives, the early to later layers in DNNs trained to learn speech representations correlate with the successive processing in the ascending auditory pathway. HG representation is not modeled well by speech DNNs (*P* > 0.1 in all layers compared to baseline; Fig. 1a), although the latencies and temporal integration windows for TRFs would suggest a serial processing pathway.

### DNN layers correlate with distinct STG populations

Previous studies have identified neural populations in the STG that show distinct speech-responsive profiles, including onset and sustained responses^34,38^. Here, we evaluated whether these functionally distinct speech-responsive populations correspond to different layers in the same DNN model.

**Fig. 3:**
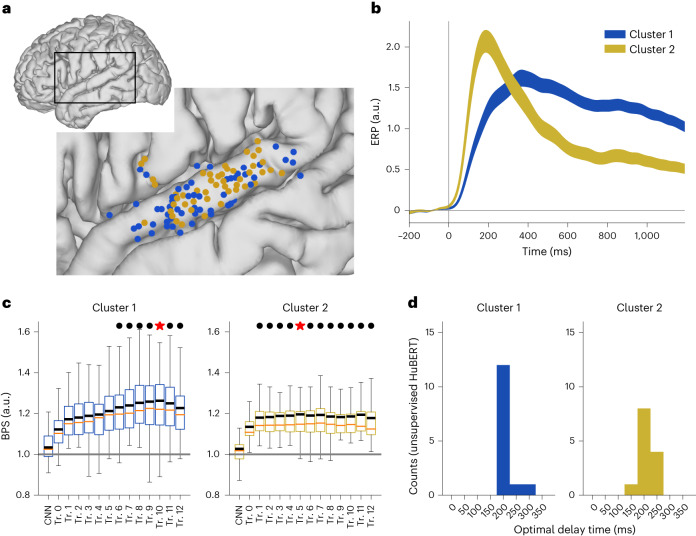
Functional subpopulations in the STG correlate with different contextual representation layers in DNNs. **a**, Anatomical locations of all speech-responsive electrodes, mapped onto a common cortical space in the enlarged image of the boxed region. Different colors indicate different functional clusters. **b**, Averaged event-related potential (ERP) of each functional cluster. All time points were aligned with sentence onsets and normalized to the resting-state baseline (mean ± s.e.m.). **c**, Normalized BPSs of the encoding models based on every single layer in HuBERT for each functional cluster (maximum over delay window lengths). Red star indicates the layer with the highest score; black dot indicates other layers that were not statistically different from the best layer (*P* > 0.05, paired *t* test, two-sided; *n* = 83 electrodes for cluster 1, *n* = 61 electrodes for cluster 2). Box plot shows the first and third quantiles across electrodes (orange line indicates the median; black line indicates the mean value; and whiskers indicate the 5th and 95th percentiles). Horizontal gray line: the performance of the full acoustic-phonetic feature baseline model. **d**, Histogram of the optimal delay windows corresponding to models in **c**. Source data

**Extended Data Fig. 6:**
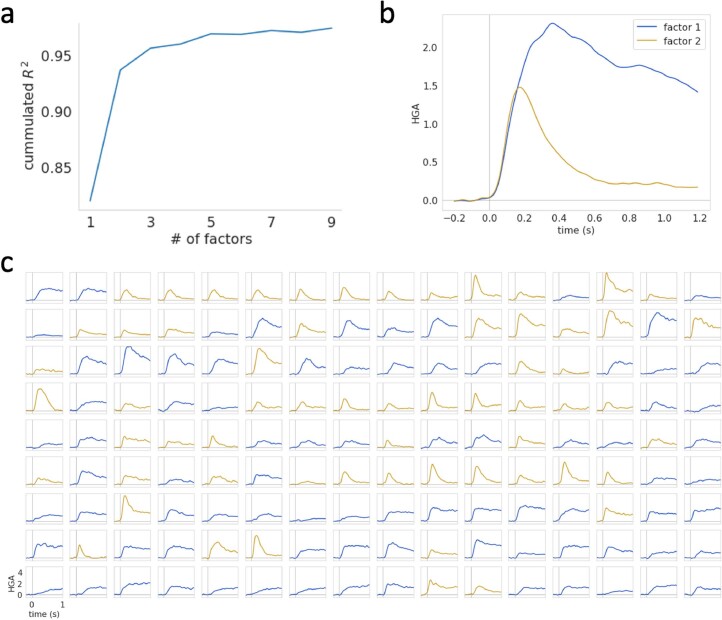
Clustering the STG electrodes. **a**) Percent of total variance explained by the NMF decomposition with different number of factors; **b**) The time course of the event-related high-gamma activity (HGA) of the two factors from the NMF model; **c**) the cluster assignment for each STG electrode. Each panel is the sentence averaged neural response for one STG electrode, colored by the cluster assignment. Source data

To identify functionally distinct populations in the STG, we performed non-negative matrix decomposition on the averaged speech-evoked response to cluster speech-responsive electrodes. Among the 144 speech-responsive electrodes in the STG, we found two clusters that showed distinct onset and sustained response profiles based on averaged high-gamma responses across sentences^38^ (Fig. 3a,b and Extended Data Fig. 6). Note that we used a slightly different clustering strategy and clustered trial-averaged responses instead of single-trial responses as in the study by Hamilton et al.^38^. We found similar onset and sustained functional populations as in Hamilton et al.’s study^38^ but not the same anatomical distinctions. However, our results align with those from Hamilton et al.’s recent study^34^, which demonstrated that the posterior STG has a concentrated transient onset response and the middle and posterior STG areas have a more distributed sustained phonetic and pitch encoding.

We then investigated the best prediction model for STG responses, the HuBERT model, and compared the BPSs of different layers with regard to the functional clusters. We found that both clusters were better explained by the contextual layers in the HuBERT model. As shown in Fig. 3c, for the more sustained cluster (cluster 1), the best prediction model came from the deep layers of the transformer encoder in the DNN (cluster 1: peak BPS = 1.26 at transformer layer 10). The deep layers of the transformer encoder performed significantly better than the early layers in the DNN (*P* < 0.05, two-sided paired *t* test; degrees of freedom (d.f.) = 83; no statistical difference across layers 6–12). For the more transient cluster (cluster 2), the best prediction model was from transformer layer 5 in the DNN (peak BPS = 1.20 at layer 5). However, the peak prediction layer did not significantly outperform any other transformer layers in the network except the very first one (*P* > 0.05 for all two-sided paired *t* tests, d.f. = 61 for cluster 2). Clusters 1 and 2 showed a similar optimal delay-time window of approximately 200–250 ms (Fig. 3d). As a result, the sustained speech-responsive neural activity prevalent in the STG can be predicted from the deeper representation layers in the DNN, whereas the more transient speech-responsive neural activity, such as the onset response, can be predicted in both the early and late parts of the transformer hierarchy in the DNN. The DNN maintains the transient onset representation throughout the processing hierarchy, and the later layers represent both transient and sustained representations in parallel. This suggests that some features, especially highly salient ones such as phrasal and sentence onsets, may be represented in multiple layers across the DNN model.

### DNN computations explain neural encoding predictions

We next examined the computational mechanism underlying representations in the DNN. We asked whether certain types of attentional computation for speech in the DNN explain the ability to predict brain responses. Here, we particularly focused on attention regarding the phonological context, which corresponds to the neighboring phonemes and syllables of the target speech sound.

Specifically, we used the HuBERT model as the target model and extracted the attention-weight matrices in each transformer layer of the DNN, which quantified the contributions from different context parts to the feature representation at each time. Critically, these contextual attention-weight matrices were not static filters but rather dynamically changed according to the specific speech sequences. Therefore, they reflect the stimulus-dependent dynamic extraction of contextual information in each speech sequence. Such computations are important for extracting the informative sequential feature representations of acoustic signals.

**Fig. 4:**
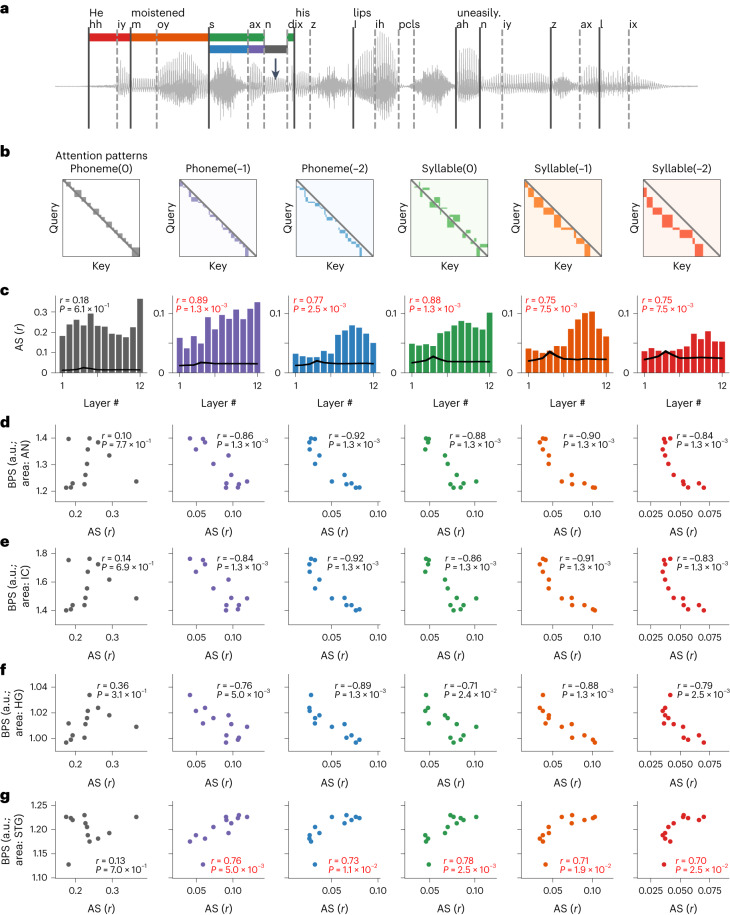
Context-dependent computations explain brain correspondence across layers in the DNN. **a**, Sample speech sentence text, waveform and phonemic annotations. The segmentations of phonemic and syllabic contexts to the current timeframe (black arrow) are marked in different colors: phoneme(0), current phoneme (gray); phoneme(−1), previous phoneme (purple); phoneme(−2), second to the previous phoneme (blue); syllable(0), current syllable (excluding the current phoneme; green); syllable(−1), previous syllable (orange); syllable(−2), second to the previous syllable (red). **b**, Template attention-weight matrices for different contextual structures as shown in **a**. ‘Query’ indicates the target sequence. ‘Key’ indicates the source sequence. Colored blocks correspond to different contexts. **c**, Averaged AS (Pearson’s correlation coefficient between attention weights and templates) across all English sentences for each transformer layer corresponding to each type of attention template. *r* values in the top left of each panel indicate the correlation between the AS and the layer index (*n* = 12 different layers, permutation test). Black line indicates the averaged AS from the same DNN architecture with randomized weights (mean ± s.e.m., *n* = 499 independent sentences). **d**–**g**, Scatter plots of AS versus BPS across layers for the AN (**d**), IC (**e**), HG (**f**) and STG (**g**) areas. Each dot indicates a transformer layer, and each panel corresponds to one type of attention pattern. The *r* and *P* values correspond to the AS–BPS correlation across layers (Pearson’s correlation, permutation test, one-sided. Red fonts indicate significant positive correlations). Source data

As a result, for each sentence in the speech corpus, we defined templates of attention matrices corresponding to different levels of contextual information representation in speech, including contextual information within the same phoneme, contextual information from the previous phoneme(s), contextual information within the same syllable and contextual information from the previous syllable(s) (Fig. 4a,b). We then computed the averaged correlation coefficient between the actual attention-weight matrices in each DNN layer and the templates across all sentences, which we termed the attention score (AS) (Fig. 4c). We found a general trend that deeper layers had an increased amount of contextual attention to linguistic structures (previous phoneme(s) and syllable(s)) (Fig. 4c, bar plots). A randomized DNN model with the same architecture but no pretraining on speech data did not show such progressive contextual attention along the hierarchy (Fig. 4c, black lines). Therefore, the alignment of attention with contextual structures not only was a direct consequence of the hierarchical architecture of the DNN model that emerges with depth but also reflected computations adapted to extracting speech-specific, linguistically relevant representations through training on natural speech (Fig. 4c).

We then tested whether such trends in contextual computations would predict the brain-prediction performance of different layers in the DNN. Specifically, we correlated the AS with the BPS for each brain area in different DNN layers. We found that the phonemic- and syllabic-level attention to the linguistic context in speech was positively correlated with the ability to predict brain activity only in the nonprimary auditory cortex (Fig. 4g) but not in the auditory periphery or the primary auditory cortex (Fig. 4d–f). In other words, for a given transformer layer in the model, the better the attention weights aligned with the linguistic contextual structure, the better the layer’s learned representation would be able to predict the speech response in the STG. Conversely, the more contextual information attended, the less the learned representation would be correlated with the AN–IC–HG response.

### DNN encoding models capture language-specific information

**Fig. 5:**
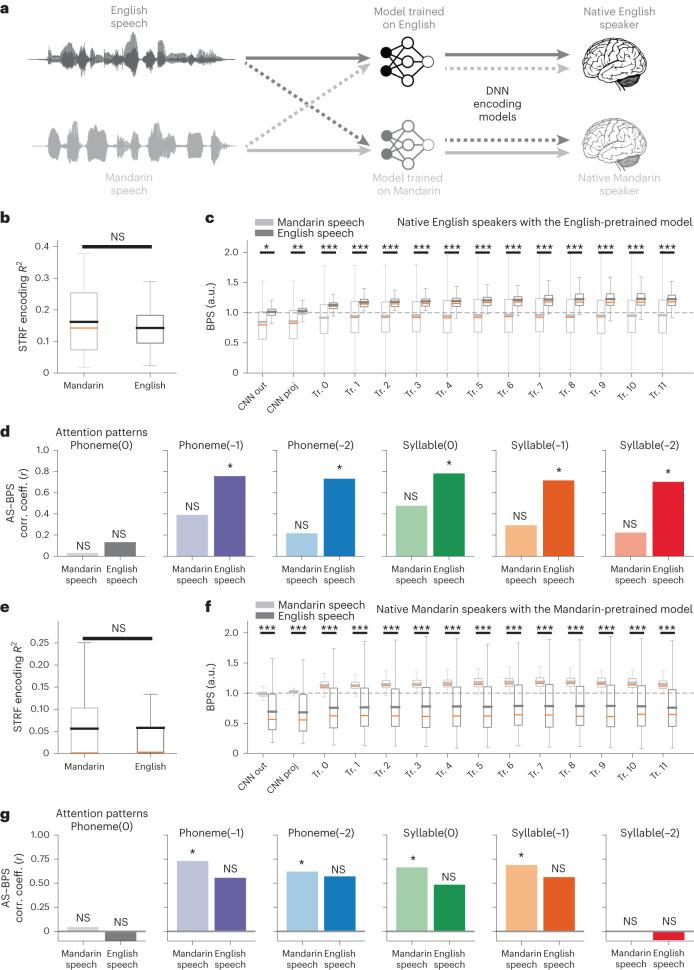
Cross-language encoding comparisons reveal language-specific representations and computations aligned between the DNN and the STG. **a**, Schematic of the cross-language paradigm. Both English (darker shade) and Mandarin (lighter shade) speech samples were fed into models pretrained on English or Mandarin. The extracted representations were used to predict neural responses recorded in the STG of native English speakers or native Mandarin speakers when they listened to the corresponding speech. **b**, Distribution of the prediction *R*^2^ values of the linear STRF model in STG electrode recordings from native English speakers using English or Mandarin speech. Two-sided paired *t* test. **c**, Averaged normalized BPS of the encoding model based on every single layer in the English-pretrained HuBERT model in native English speakers when they listened to English versus Mandarin speech. *P < 0.05, **P < 0.01, ***P < 0.001, paired two-sided *t* test; *n* = 57 electrodes in the STG (a subset of all participants who completed the relevant tasks). **d**, AS–BPS correlation across layers in the English-pretrained HuBERT model and the STG in native English speakers (Pearson’s correlation, *P < 0.05, permutation test, one-sided). Each panel corresponds to one type of attention pattern. Colored bars correspond to different contexts, as in Fig. 4. **e**–**g**, Same as **b**–**d** but using the Mandarin-pretrained HuBERT model and recordings from *n* = 61 STG electrodes in native Mandarin speakers. Box plot shows the first and third quantiles across electrodes (orange line indicates the median; gray line indicates the mean value; and whiskers indicate the 5th and 95th percentiles). Dashed horizontal gray line: the performance of the full acoustic-phonetic feature baseline model. CNN out, CNN output layer; CNN proj, CNN projection layer; NS, not significant. Source data

Next, we tested whether DNN computations and representations are language specific and reflect higher-level language processing beyond the acoustics, such as phonotactic, phonological or lexical representations. To do this, we used a cross-linguistic approach by comparing English and Mandarin (Fig. 5a). Mandarin shares many consonants and vowels with English but largely differs in how phonetic and prosodic features are combined to give rise to words. In addition to data from English-speaking participants, we also analyzed cortical recordings from three native Mandarin speakers (Extended Data Fig. 1). Both groups were monolingual and had no comprehension of the foreign language. We adopted the same paradigm and materials as our previous study that focused on cross-linguistic pitch perception^39^. The two participant groups were instructed to listen to both naturalistic English speech and Mandarin speech in separate recording blocks. In addition to the previous HuBERT model pretrained on English speech, we also pretrained the same HuBERT model on naturalistic Mandarin speech. We then compared the performance of the two HuBERT models on the two groups when they listened to different languages (Fig. 5a).

To explicitly test our hypotheses of linguistically relevant, context-dependent processing in the auditory pathway as shown in the previous section (Fig. 4), we conducted cross-lingual perception and DNN prediction tests. In particular, we hypothesized that the contextual-dependent computations in the DNN capture language-specific, higher-level processing beyond the acoustics in the STG. Therefore, we expected the English-pretrained model to show higher brain-prediction performance for the STG in native English speakers and that the prediction performance would be better aligned with contextual attention to the phonemic and syllabic structures in English than in Mandarin. On the contrary, we expected the Mandarin-pretrained model to show higher brain-prediction performance and better correlation with contextual attention for Mandarin speech in native Mandarin speakers.

First, we examined the results with an English-pretrained model and native English speakers. At the acoustic level, the linear spectrogram TRF (STRF) model, which included only spectrogram features, showed similar performance in predicting neural responses in the STG when the participants listened to different languages (mean *R*^2^ = 0.162 and 0.143 for Mandarin and English speech, respectively; paired *t*(57) = 1.65, *P* = 0.104, two-sided; Fig. 5b). This suggests that lower-level acoustic representations are largely shared across languages. However, a performance gap was found in the DNN encoding models between languages, in which the BPS for English speech was significantly higher than that for Mandarin speech (13 of 14 comparisons had *P* < 0.01, paired *t* test, two-sided; Fig. 5c). Moreover, the gap between the two languages monotonically increased in deeper layers of the network: ΔBPS = 0.160 at the CNN output layer (paired *t*(57) = 2.55, two-sided *P* = 0.013), ΔBPS = 0.211 at the first transformer-encoder layer (paired *t*(57) = 3.20, two-sided *P* = 0.002) and ΔBPS = 0.314 at the tenth transformer-encoder layer (paired *t*(57) = 4.56, two-sided *P* = 3 × 10^−5^) (Fig. 5c). This suggests that the representation in the network demonstrates an increasing level of language-specific information. We also evaluated the relationship between the computation of phonemic and syllabic contextual information in DNN layers and the corresponding brain-prediction performance for Mandarin speech in the STG. As opposed to previous results (Fig. 4g), no significant correlation was found in either the phonemic or syllabic level between the attention patterns in DNN layers and the BPSs when native English speakers listened to Mandarin speech (*P* > 0.05 for all cases, permutation test; Fig. 5d).

In contrast, we found opposite results with a Mandarin-pretrained model and native Mandarin speakers. At the acoustic level, the linear STRF model also showed similar performance for both Mandarin and English speech (mean *R*^2^ = 0.056 and 0.058 for Mandarin and English speech, respectively; paired *t*(61) = −0.501, *P* = 0.617, two-sided; Fig. 5e). The DNN encoding models showed consistently higher performance for neural responses to Mandarin speech than English speech (all 14 of 14 comparisons had *P* < 0.01, paired *t* test, two-sided; Fig. 5f), and the gap also increased in deeper layers: ΔBPS = 0.293 at the CNN output layer (*P* = 6 × 10^−7^, paired *t*(61) = 5.57, two-sided) and ΔBPS = 0.405 at the ninth transformer-encoder layer (*P* = 6 × 10^−9^, paired *t*(61) = 6.76, two-sided; Fig. 5f). Moreover, as opposed to the combination of the English-pretrained model and native English speakers, we found consistently significant correlations between phonemic- or syllabic-level ASs and BPSs when listening to Mandarin speech (*P* < 0.05, permutation test), and no significant correlation when listening to English speech, in these native Mandarin speakers (*P* > 0.05 for all cases, permutation test; Fig. 5g).

Therefore, our results demonstrate a double-dissociation pattern between pretrained models and native languages, suggesting that DNN computations and representations capture higher-level, language-specific linguistic information in the STG that is learned depending on language experience.

### DNN acoustic–phonetic hierarchy explains brain prediction

The last question we asked is whether the brain-prediction performance of the DNN layers can be accounted for by an acoustic-to-phonetic processing hierarchy. We tested the feature representations of acoustic, phonetic and prosodic information in the DNN layers. Specifically, we applied similar linear feature-encoding models to predict the activations of hidden units in different DNN layers and computed the unique variance explained by each set of features. These features are statically coded and do not vary according to different contexts. Therefore, our analysis here intentionally reflects the static noncontextual part of acoustic/phonetic/prosodic representations in DNN layers, as addressed in the previous analyses.

**Fig. 6:**
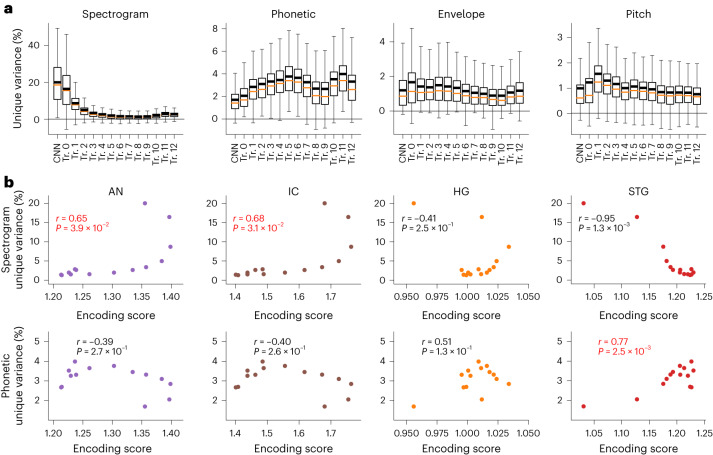
Representations in neural networks demonstrate an acoustic-to-phonetic transformation hierarchy yet preservation of prosodic cues through DNN layers. **a**, Distribution of the unique variance explained by each set of features across units in each DNN layer. *n* = 512 units in the last CNN layer and 768 units in each transformer layer. Box plot shows the first and third quantiles across electrodes (orange line indicates the median; black line indicates the mean value; and whiskers indicate the 5th and 95th percentiles). **b**, Top row, correlation between the BPS and the unique variance explained by spectrogram features in each layer; bottom row, correlation between the BPS and the unique variance explained by phonetic features in each layer. Each panel corresponds to one area, with each area represented by a different color (*n* = 14 layers, two-sided *t* test). Red fonts indicate significant positive correlations. Source data

Overall, the results demonstrated an acoustic-to-phonetic transformation along the hierarchy (Fig. 6a). In the CNN output layer, acoustic (spectrogram) features uniquely accounted for 20.0% of the total variance, whereas phonetic features accounted for only 1.70% (paired *t*(768) = 47.6, *P* < 1 × 10^−10^, two-sided). However, after the third transformer encoder, phonetic features consistently explained more unique variance than the acoustic features in the network (3.45% versus 2.66% at Tr. 4 for phonetic and acoustic features respectively, paired *t*(768) = 5.77, *P* = 5.7 × 10^−9^). The unique variance explained by static phonetic features peaked at the 11th transformer-encoder layer with a unique *R*^2^ of 3.98% (paired *t*(768) = 9.12, *P* < 1 × 10^−10^, two-sided *t* test against acoustic features, which accounted for 2.85%). Meanwhile, temporal landmark (envelope) features (for example, speech envelope and onsets) and prosodic pitch features (absolute and relative pitch) were more uniformly distributed along the hierarchy of the network (Fig. 6a).

Furthermore, when correlated with the BPS of individual layers, spectrogram feature encoding showed a significant positive correlation only in the peripheral areas (AN: Pearson’s *r* = 0.65, *P* = 0.039, permutation test; IC: Pearson’s *r* = 0.68, *P* = 0.031, permutation test; Fig. 6b). Phonetic feature encoding correlated with the BPS in the STG (Pearson’s *r* = 0.77, *P* = 0.0025, permutation test; Fig. 6b) but not in the other areas (*P* > 0.05 for all of the other three areas, permutation test; Fig. 6b). Taking these together, a similar acoustic-to-phonetic hierarchy was found and correlated with both the self-supervised DNN model and the ascending AN–IC–STG pathway.

## Discussion

We have demonstrated that speech representations learned in state-of-the-art DNNs resemble important aspects of information processing in the human auditory system. DNN feature representations significantly outperform theory-driven acoustic–phonetic feature sets in predicting neural responses to natural speech throughout the auditory pathway. DNN-layer hierarchy correlates with the AN–midbrain–STG ascending auditory pathway. Deeper DNN layers correlate with functionally distinct speech-tuned populations in the nonprimary auditory cortex. We inspected the core contextual computations in DNNs and found that they learn critical linguistically relevant temporal structures, such as phoneme and syllable contexts, from purely unsupervised natural speech training. Such ability to learn language-specific linguistic information predicts DNN–neural coding correlation in the nonprimary auditory cortex. DNN-based neural encoding models can reveal language-specific coding in the STG during cross-language perception, whereas linear STRF models cannot.

### DNN models reveal important neural coding properties in the speech–auditory cortex

**Extended Data Fig. 7:**
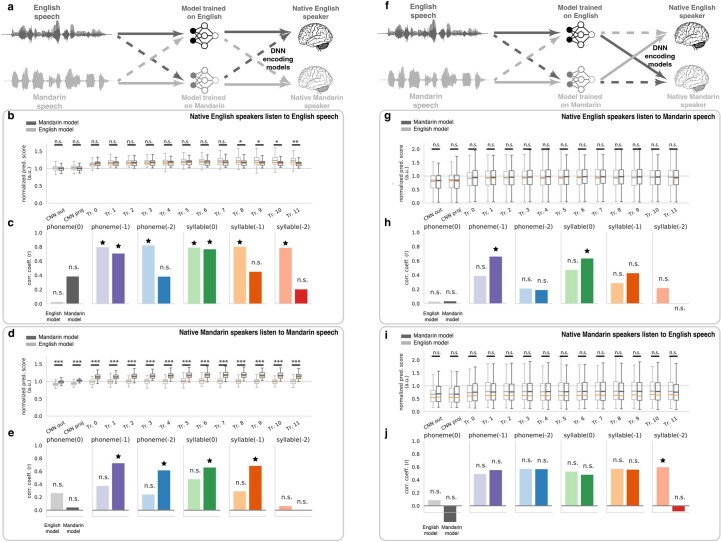
Cross-model encoding comparisons reveal language-specific representation and computations aligned between DNN and STG. **a)** Schematic of the cross-model paradigm. Both English (lighter color) and Mandarin (darker color) speech were fed into models pretrained on English or Mandarin. The extracted representations were used to predict neural responses recorded in STG from native English speakers or native Mandarin speakers when they listened to the corresponding speech (English speaker listened to English; Mandarin speaker listened to Mandarin). **b)** The distribution of normalized brain prediction score of the encoding model based on every single layer in English-pretrained HuBERT model (light shaded bars) versus Mandarin-pretrained model (dark shaded bars) in native English speakers when listening to English speech. * *p* < 0.05, ** *p* < 0.01, *** *p* < 0.001, paired two-sided t-test; n = 57 electrodes in STG. **c)** The AS-BPS correlation across layers in English-pretrained (light shaded bars) and Mandarin-pretrained (dark shaded bars) HuBERT model with STG in native English speakers (Pearson’s correlation, * *p* < 0.05, permutation test, one-sided). Each panel corresponds to one type of attention pattern. (See also Fig. 4). **d-e)** Same as **b-c**, but using recordings from STG in native Mandarin speakers when listening to Mandarin speech (n = 61 electrodes in STG). The performance of English-pretrained model (light shaded bars) and Mandarin-pretrained HuBERT models (dark shaded bars) are compared. **f-j)** same as **a-e**, but for native English speakers or native Mandarin speakers when they listened to speech in the other language (English speaker listened to Mandarin; Mandarin speaker listened to English). Box plot shows the first and third quantiles across electrodes, orange line indicates the median, gray line is the mean value, and whiskers indicate the 5th and 95th percentiles. Source data

Encoding models are prevalent methods to approach the neural coding of sensory perception^10,30,40^. Despite achieving success with lower-level acoustic–phonetic features^10,34,41–43^, linear encoding models struggle with higher-order speech information, often failing to reveal information beyond acoustic stimulus encoding (Fig. 5b,e). Previous studies using activation contrasts or linear models have not found the language-specific contextual effects of acoustic–phonetic coding in local populations in the STG^39,44,45^, but DNN-based representations detect such language-specific coding in single STG electrodes (Fig. 5 and Extended Data Fig. 7). To account for nonlinear transformations of pure acoustic cues in the auditory system, studies have included higher-order features, such as phonetic, phonemic, syllabic and lexical features, as predictors^34,39,46,47^. However, these feature representations rely on strong presumptions of hierarchical neural coding of these exact divisions, potentially missing intermediate representations in the nonprimary auditory cortex^48,49^. Furthermore, these models posit the auditory system as a passive finite response filter, neglecting the prevalent non-onset recurrent activity in higher-order speech areas^34,48,50^.

Traditional hierarchical models of neurobiology suggest that specific brain areas specialize in distinct representation levels and information is transformed in anatomically defined ‘streams’ (that is, sound to phoneme to syllable to word and semantics)^9,51^. Our results challenge this traditional view. Although we observed a transformation from spectrogram to phonetic features, instead of phonemes and syllables as discretely encoded representations, we found complex, distributed higher-order representations that also carry forward prosodic information that may originate at earlier auditory levels and that processing is highly context dependent in later layers of computation. These findings explain the existence of both phonetic-feature tuning^42^ and diverse ‘lower-level’ (onset, peak rate, frequency tuning)^34,46^ and ‘higher-level’ (context dependence, normalization, lexical effects) representations in the STG^39,47,52,53^.

### DNNs as computation models of the auditory pathway

Our results highlight two critical factors behind DNN models’ superior performance over heuristic linear models with static speech features: (1) DNN model nonlinearity—almost all DNN layers consistently outperformed feature TRF models, even in the auditory periphery. This is consistent with demonstrations of nonlinear processing in the auditory periphery^54^. Despite comparable amounts of predictors (on the order of 10^2^), DNNs learn nonlinear features for better speech representations. (2) DNN models’ dynamic temporal integration of phonological contextual information—this is especially pivotal for higher-order speech responses in the nonprimary auditory cortex. STG responses were better predicted using deeper DNN layers with extended delay-time windows. Simply using static nonlinear filters in CNN layers with an even longer delay-time window could not achieve similar prediction performance for STG responses (Fig. 2). This indicates that specific dynamic temporal integration, aligned with the contextual information in speech and parametrized by computation models such as transformers or recurrent neural networks, is critical for characterizing STG speech responses. Dynamic contextual computations are also correlated with higher-level language processing in the cortical language network^55^. Our findings suggest that the STG processes speech at dynamic timescales, possibly underpinning temporal binding of phonological sequences to form dynamic acoustic–phonetic and ultimately perceptual representations of speech^50^.

Our results offer new insights into computations in the auditory pathway. In DNN models, model architecture determines the computation and representation capacity^56^. We found that different computational architectures better correlate with different parts of the auditory pathway: the convolution layers in DNNs are apt for the auditory periphery and subcortical areas with locally resolved static nonlinear filters; deeper transformer-encoder and LSTM layers better fit the speech–auditory cortex, with more complex stimulus-dependent temporal dynamics than static spectrotemporal filters. These computational attributes emerge as signatures for respective parts of the auditory pathway: the auditory periphery and subcortical structures are characterized by ascending feedforward synaptic connections for rapid forward-filtering of signals^32^, whereas the speech–auditory cortex has a multilayer architecture with reciprocal connections facilitating sustained computations similar to recurrence and attention^57^. In contrast to prior cortex-centric studies, our study reveals speech-relevant computations spanning the entire auditory pathway through the lens of DNNs for speech representation learning.

This has major implications for interpreting the functions of the primary and nonprimary auditory cortical areas. Dynamic computations and representations showed limited contribution to predicting speech responses in the primary auditory cortex beyond the static convolutional filters (Figs. 2 and 4). In contrast, prediction of sustained STG responses to speech strongly correlated with dynamic computations in DNNs (Figs. 2–4). This discrepancy aligns with a recent study highlighting distinct phonological and complex sound processing in the STG versus tonotopic, narrow-tuned sound processing in the primary auditory cortex^34^. The STG also receives direct thalamic inputs through the nontonotopic, nonlemniscal pathway^58–60^ and does not appear to be solely dependent on the primary auditory cortex^61^. Our findings challenge the primary auditory cortex’s sole contribution to advanced computational models of speech processing, despite previous assumptions that it causally functions like the primary visual cortex in object recognition processing within the ventral stream^51,62^. Notably, we should also point out that, owing to limited experiment time during awake surgeries, we did not evaluate the cross-language question regarding the HG in this study.

### Self-supervised seq2seq learning and the speech–auditory cortex

Our results demonstrate that self-supervised DNNs match or exceed the performance of more prevalent supervised models in predicting brain responses to speech. The training objective critically shapes DNN representations. Previous works have found that supervised discriminant learning, such as word classification^17,18^, leads to feature representation correlating with auditory neural responses. Our results are consistent with these findings. However, instead of using a discrete classification task, we show that a specific type of supervised seq2seq learning task, ASR, induces neurally correlated speech features. Furthermore, self-supervised learning, including contrastive and predictive learning, similarly produces matching representations aligning with STG responses to speech. For naturalistic speech perception, previous studies do not support discrete selective coding for word forms in the STG but rather a collection of local populations tuned to complex acoustic–phonetic cues and temporal landmarks in speech^34,50,53,63^. Therefore, a single supervised task such as word decoding may not capture all computations and representations in the STG. Meanwhile, self-supervised learning yields richer representations beyond the requirement of pure speech recognition, such as prosodic information and speaker identity. Our results show that fine-tuning supervised ASR tasks on top of the unsupervised pretraining does not further improve the overall brain encoding performance in the STG. Conversely, we observed that the brain-prediction performance for the nonprimary auditory cortex decreased in the deep layers after supervised fine-tuning (Extended Data Fig. 5).

From a computational modeling perspective, our results extend previous successes in using DNNs as models of sensory systems^21,64^. Recent studies have adopted end-to-end training of DNNs to predict neural responses^65,66^. Although this approach directly optimizes brain-prediction performance, a considerable amount of data is required for training. For instance, the seq2seq DNN models we used here have approximately 100 million parameters and were trained on ~1,000 h of speech for competitive performance^13–15^. Collecting an equivalent amount of neural data is unfeasible within our clinical settings. Furthermore, owing to the nature of intracranial recordings, only a sparse sample (~100 electrodes) from the auditory cortex was available for each participant. As a result, the learned representations from a straight end-to-end optimization of brain activity may be biased by the individual difference in electrode sampling. Instead, we used a transfer learning paradigm, pretraining DNNs without any neural data as inputs, and demonstrated that speech representations learned by these DNN models are also transferable to the neural coding process in the auditory pathway. Importantly, the DNNs used in this study were all trained on a completely independent dataset from the one used for neural recordings. Moreover, unsupervised models abstain from explicit speech information or linguistic knowledge. Unlike classical computational models of speech perception, such as TRACE^9^, assuming a strict acoustic–phonetic–lexical hierarchy and explicit top–down inference, our pure data-driven self-supervised models yield an emerging acoustic–phonetic hierarchy. The self-supervised models’ analogous representation hierarchy to the human auditory system suggests that the two systems may share similar computations that extract critical statistical structures of speech.

Our results extend the current literature on using task-optimized pretrained DNN models to predict cortical auditory responses. Compared to the previous pioneering study by Kell et al., which mainly used fMRI recordings and CNN models pretrained on tasks such as word recognition^17,64^, our study offers new insights from models with different architectures and computational objectives. Coupled with use of intracranial electrophysiological recordings with high temporal resolution, our approach allows for analysis of dynamic temporal coding of speech as a rapidly time-varying signal. We also show hierarchical processing, as reported in previous studies; however, our results show that early processing also occurs in subcortical pathways.

**Extended Data Fig. 8:**
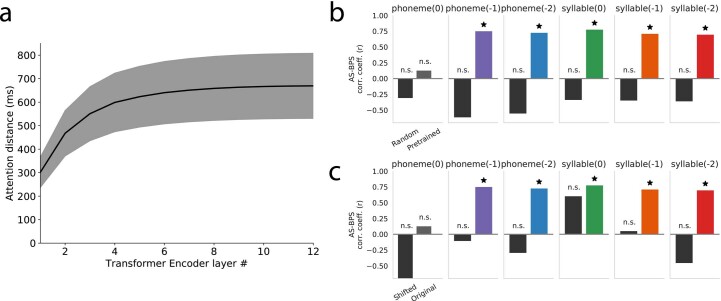
Analysis on attentions in HuBERT model. **a)** The averaged attention distance in each Transformer encoder layer of HuBERT model (mean ± s.d., n = 499 independent sentences). The averaged attention distance is computed as token distance weighted by attention weights, averaging across all attention heads and across all tokens. The attention weights in each layer are iteratively aggregated over previous layers using attention rollout. **b)** The AS-BPS correlation across layers in random model versus English-pretrained model for STG in native English speakers (Pearson’s correlation, * *p* < 0.05, permutation test, one-sided). Each panel corresponds to one type of attention pattern. (See also Fig. 4). **c)** The shifted AS-BPS correlation (with attention matrix shuffled in blocks) across layers versus unshifted original AS-BPS in English-pretrained model for STG in native English speakers (Pearson’s correlation, * *p* < 0.05, permutation test, one-sided). Each panel corresponds to one type of attention pattern. Source data

Modern DNN models are complex dynamic systems influenced by factors such as architectures, hyperparameters and optimization procedures. Hierarchical CNNs deterministically enforce receptive field growth across layers; however, transformer encoders have no prior constraint on the hierarchy of temporal context—each attention head in each layer can extend attention to the entire sequence. Therefore, the ascending patterns of contextual attention in DNNs (Fig. 4c and Extended Data Fig. 8) are learned through data-driven optimization, reflecting intrinsic, speech-aligned computations. We have established a correlation between linguistically relevant attention and neural encoding model performance. Future research remains to be done to identify other potential factors and build causal links between specific DNN computations and brain encoding.

### Limitations

Our results suggest how different levels of speech representations emerge from hierarchical bottom–up recurrent or self-attentional operations and how these representations correlate with the auditory cortex. Omitted are top–down modules and cortical areas beyond the auditory cortex, such as the frontal areas. Therefore, it remains to be delineated how other areas in the language network interact with the auditory cortex, whether these interactions modulate local and populational representations of speech, and to what extent these interactions can be characterized by our proposed framework. Besides coverage, our analysis focused on the temporal dynamics within individual electrodes. Future work should address how DNN feature representations align with distributed population-level neurodynamics^67^ in the auditory cortex.

A potential limitation concerns the biological plausibility of the computational models used in this study. The transformer and LSTM models considered in this study are bidirectional and noncausal. This would complicate the analysis of precise temporal dynamics in speech sequences. We focused on learned feature representations rather than actual parametrizations and implementations of algorithms such as self-attention or the LSTM mechanism. We cannot assert that any of these computations are implemented in the cortex or that gradient-based learning mirrors brain mechanisms. Despite correlational evidence, formal fine-grained causal and ablation analyses remain to be conducted to investigate the detailed relationship between computational components in DNNs and model-predicted neural responses. However, it is promising that in silico models converge on a similar representational basis of speech as the brain, with a learning algorithm that does not require millions of labeled examples and is a potentially strong candidate for a biologically plausible theory of sensory learning^26^ or higher-level language processing in general^27^.

**Extended Data Fig. 9:**
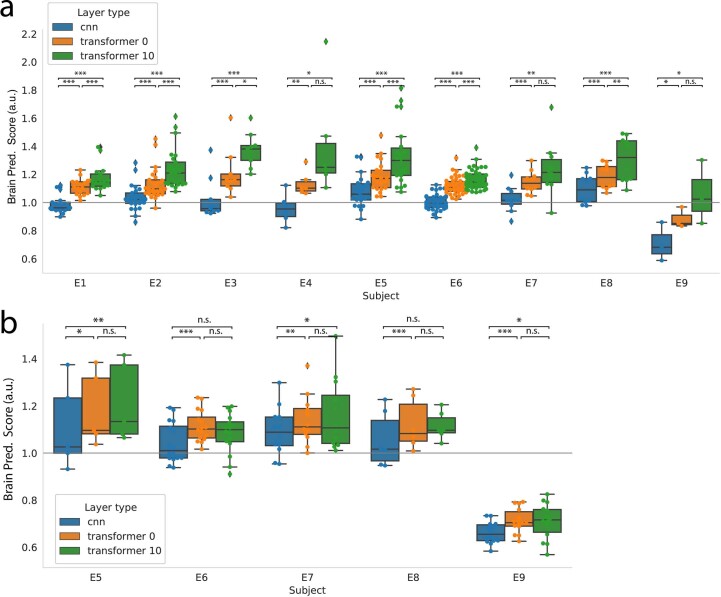
Comparing DNN encoding performance for STG and HG in individual subjects. This is supplement to Fig. 2a. The averaged normalized brain prediction score on single layer encoding models in the HuBERT model (maximum over delay window length). Three representative layers are used: the CNN output, the first Transformer layer, and the 10^th^ Transformer layer (the optimal layer shown in Fig. 2). **a)** E1-E9 are the nine native English speakers. Each dot in the swarm plot represents one single electrode in STG (only speech responsive electrodes are plotted). Box plots show the 25/50/75 quantiles, whiskers indicate the 5th and 95th percentiles. The black statistical significance markers are determined using two-tailed paired t-test between different layers (* *p* < 0.05, ** *p* < 0.01, *** *p* < 0.001, n.s. *p* > 0.05, n = 17, 25, 9, 6, 22, 42, 10, 10, 3 individual electrodes.). **b)** Same as **a**, for the five participants with HG coverage (E5-E9); n = 5, 16, 12, 6, 14 individual electrodes. Source data

**Extended Data Fig. 10:**
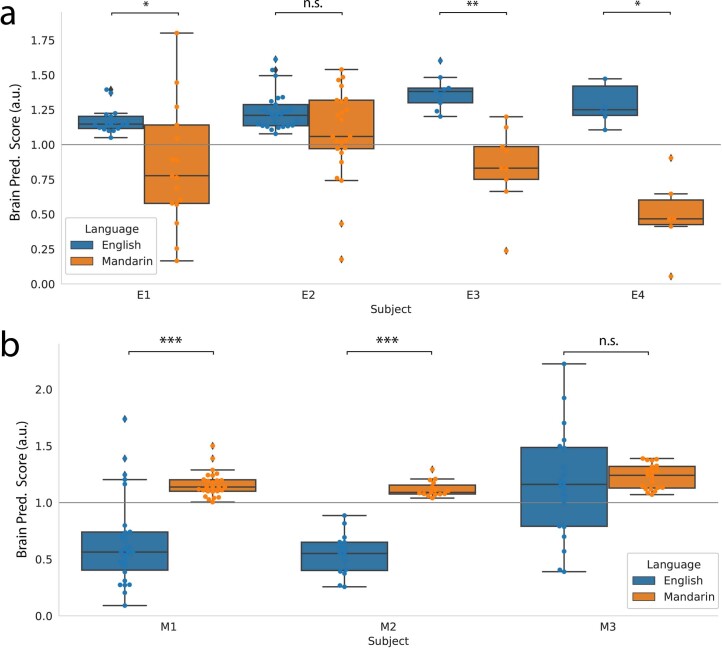
Comparing DNN encoding performance across different languages in STG of individual subjects. This is supplement to Fig. 5c. **a)** The distribution of normalized brain prediction score on single layer encoding models in the English-pretrained HuBERT model (maximum over delay window length) over individual STG electrodes. The 10th Transformer layer (the optimal layer shown in Fig. 2) is used. E1-E4, M1-M3 are the four native English speakers and three native Mandarin speakers that listen to both English and Mandarin speech. Each dot in the swarm plot represents one single electrode in STG (only speech responsive electrodes are plotted). Box plots show the 25/50/75 quantiles, whiskers indicate the 5th and 95th percentiles. The black statistical significance markers are determined using two-tailed paired t-test between different layers (* p < 0.05, ** p < 0.01, *** p < 0.001, n.s. p > 0.05; n = 17, 25, 9, 6 individual electrodes). **b)** Same as **a**, for Mandarin speakers M1-M3, n = 26, 15, 20 individual electrodes. Source data

Owing to the relatively small number of participants tested, our statistical analyses were performed across electrodes and did not consider between-participant variability, thereby lacking interindividual generalization across the population. This limitation is common in intracranial studies and outweighed by the unique opportunity to record intracranially from human patients. Nonetheless, our results were largely consistent across participants (Extended Data Figs. 9 and 10). Future research could explore and validate these findings in larger and more diverse populations, as well as with a broader spectrum of AI models.

## Conclusion

Using a comparative approach, we show important representational and computational parallels between speech-learning DNNs and the human auditory pathway. From a neuroscientific perspective, data-driven computational models excel in extracting intermediate speech features from statistical structures, surpassing traditional feature-based encoding models. From the AI perspective, we unveil an avenue to understand the ‘black box’ representations in DNNs by comparing them to neural responses and selectivity. We show that modern DNNs may have converged on representations that approximate processing in the human auditory system.

## Methods

The experimental protocol was approved by the institutional review boards at the University of California, San Francisco (UCSF), and Huashan Hospital, Fudan University. All participants provided written informed consent before undergoing testing. All patient data were stored and analyzed on computing servers within UCSF, and Meta AI Research performed DNN model pretraining using publicly available speech corpora, without access to patient data.

### Participants

This study included 12 monolingual participants (6 men and 6 women, aged 31–55 years, all right-handed) who were neurosurgical patients at either the UCSF Medical Center or Huashan Hospital. No statistical methods were used to predetermine sample sizes, but our sample sizes are similar to those reported in previous publications^34,39,42,47,52^. Nine native English-speaking participants from UCSF (E1–E9) were either eloquent patients with brain tumors (four patients) undergoing awake language mapping as part of their surgery or patients with intractable epilepsy (five patients) implanted with high-density electrode grids for clinical monitoring of seizure activity (all with left-hemisphere coverage). We included only participants with tumors that had not invaded the auditory cortex. Three native Mandarin-speaking participants from Huashan Hospital (M1–M3) were eloquent patients with brain tumors undergoing awake language mapping as part of their surgery (all with left-hemisphere coverage). The placements of the grids were determined solely by clinical needs. All patients were informed (as detailed in the institutional review board-approved written consent document signed by the participants) that their participation in scientific research was completely voluntary and would not directly affect their clinical care. Additional verbal consent was also acquired at the beginning and during the breaks of each experimental session. Data collection and analysis were not performed blind to the conditions of the experiments. No participants were excluded from the analyses.

### Experimental paradigm

During the experiments, the participants were instructed to passively listen to continuous speech stimuli. No other task was performed during passive listening. The acoustic stimuli used in this study consisted of natural, continuous speech in both American English and Mandarin. The English speech stimuli consisted of materials from the TIMIT dataset^36^. The TIMIT set consisted of 499 English sentences selected from the TIMIT corpus, spoken by 402 different speakers (286 male and 116 female speakers). The sentences were separated by 0.4 s of silence. The task was divided into five blocks, with each block lasting ~5 min. The Mandarin speech stimuli were a subset of the Annotated Speech Corpus of Chinese Discourse (ASCCD) from the Chinese Linguistic Data Consortium^68^, which included read texts of a variety of discourse structures, such as narrative and prose. The stimulus set consisted of 68 passages of Mandarin speech selected from the ASCCD corpus, spoken by ten different speakers (five male and five female speakers). The length of a single passage varied between 10 and 60 s. The passages were separated by 0.5 s of silence. The task was divided into six blocks, with each block lasting ~5 min.

Depending on their clinical conditions, all participants finished 3–11 blocks of all tasks. In particular, eight English-speaking participants (E1–E8) completed all five TIMIT blocks; E9 completed three TIMIT blocks; and the three Mandarin-speaking participants (M1–M3) completed two TIMIT blocks. Three English-speaking participants (E1–E3) and all three Mandarin-speaking participants (M1–M3) completed all six ASCCD blocks. E4 completed five ASCCD blocks.

### Data acquisition and preprocessing

In all patients, the same types of high-density ECoG grids (manufactured by Integra or PMT) with identical specifications (4-mm center-to-center spacing and 1.17-mm exposed contact diameter) were placed on the lateral surface of the temporal lobe. Depending on the exact clinical need, the grid may have 32 (8 × 4), 128 (16 × 8) or 256 (16 × 16) contact channels in total. In four patients (E6–E9), an additional 32-channel (8 × 4) grid with 4-mm center-to-center spacing and 1.17-mm exposed contact diameter (Integra) was placed on the temporal plane in each patient. During experimental tasks, neural signals were recorded from the ECoG grids using a multichannel amplifier optically connected to a digital signal processor (Tucker-Davis Technologies). TDT Synapse software was used for data recording. The local field potential at each electrode contact was amplified and sampled at 3,052 Hz. The raw voltage waveform was visually examined, and channels containing signal variations too low to detect from noise or continuous epileptiform activity were removed. Time segments on remaining channels that contained electrical or movement-related artifacts were manually marked and excluded. The signal was then notch-filtered to remove line noise (at 60, 120 and 180 Hz for English-speaking participants and 50, 100 and 150 Hz for Mandarin-speaking participants) and rereferenced to the common average across channels sharing the same connector to the preamplifier.

The analytic amplitude of eight Gaussian filters (center frequency 70–150 Hz) was computed using the Hilbert transform. The high-gamma signal was taken as the average analytic amplitude across these eight bands. The signal was downsampled to 100 Hz. The tasks were divided into recording blocks of ~5-min length. The high-gamma signal was *z*-scored across the recording block.

### Electrode localization

For chronic monitoring cases, electrodes were localized by aligning preimplantation MRI scans and postimplantation computed tomography scans. For awake cases, high-density electrode grids were temporarily placed onto the temporal lobe during surgery to record local cortical potentials. The three-dimensional positions of the corners of the grid were recorded using a Medtronic neuronavigation system and then aligned with the preoperative MRI scan. Intraoperative photographs were used as references. The remaining electrodes were localized by interpolation and extrapolation from those points^69^.

### Data analysis software

All analyses were carried out using custom software written in Python and MATLAB. Custom MATLAB code was used for data preprocessing. The open-source scientific Python packages that we used included PyTorch, Fairseq, HuggingFace Transformers, NumPy, SciPy, pandas, librosa and scikit-learn. Cortical surface reconstruction was performed using FreeSurfer, and electrodes were coregistered using the Python package img-pipe. Praat^70^ was used to extract pitch features. Figures were created with Matplotlib and Seaborn in Python.

### Biophysical models for the auditory periphery and midbrain

We used neuronal models of the midbrain and auditory periphery^31–33^. They consisted of a phenomenological model of AN responses, with nonlinear properties such as rate saturation, adaptation and synchrony capture, and an extended same-frequency inhibition–excitation model of the IC, which included both band-pass and low-pass/band-reject IC cells. The synaptic outputs from 50 AN neurons with characteristic frequencies uniformly distributed on a log scale within 150–8,000 Hz were extracted as the AN signal. These synaptic-output signals were used as inputs to the two different types of midbrain neurons in the IC area, which resulted in 50 band-pass IC neurons and 50 low-pass/band-reject IC cells.

For each speech sentence, the raw waveform was sent into the model as the input, and the corresponding response sequences from AN and IC cells were extracted and downsampled to 100 Hz to match the high-gamma signals from the cortex.

### Definitions of acoustic, phonetic and prosodic features

We used a heuristic set of 208 features as the baseline prediction model (161 spectrogram, 13 phonetic, 31 pitch/prosodic and 3 envelope features).

The spectrogram features of speech were calculated using a short-time Fourier transform, with 161 frequency components ranging from 0 to 8 kHz in log scale.

The phonetic features were 13-dimensional binary time series similar to those in previous works^34,42^. These features describe single phonemes as a combination of places of articulation (dorsal, coronal, labial), manners of articulation (plosive, fricative, nasal) and voicing of consonants, as well as the place of the vowel (high, mid, low, front, back) and indicator of consonant/vowel.

Pitch features, including absolute pitch, speaker-normalized relative pitch and pitch change, were extracted in the same way as in our previous work^39^. We also extracted a binary variable indicating when pitch values were present, suggesting voicing in the speech. The fundamental frequency (*F*_0_) was calculated using the autocorrelation method in Praat and corrected for halving and doubling errors. Absolute pitch was defined as the natural logarithm of *F*_0_ values in hertz. Relative pitch was computed by *z*-scoring the absolute pitch values (log(*F*_0_)) within each sentence/passage (within-speaker). Pitch change was computed by taking the first-order derivative (finite difference) in time for log(*F*_0_). We discretized absolute pitch, relative pitch and pitch change into ten bins equally spaced from the 2.5th percentile value to the 97.5th percentile value. The bottom and top 2.5% of the values were placed into the bottom and top bins, respectively. As a result, absolute pitch, relative pitch and pitch change were represented as three 10-dimensional binary feature vectors. For nonpitch periods, these feature vectors would all have a value of zero for all dimensions.

Envelope features included intensity, sentence onset and peak rate. Intensity is a continuous scalar sequence representing the envelope of speech. Sentence onset is a binary feature with a value of 1 at the onset of the first timestamp of the first phoneme in each sentence and 0 elsewhere. Peak rate was computed as previously described^46^ (that is, using a sparse time series of local peaks extracted from the first-order derivative of the amplitude envelope of speech).

### Encoding models

We used time-delayed linear encoding models known as TRF models^10^. TRF models allow us to predict neural activity based on stimulus features in a window of time preceding neural activity. In particular, we fit the linear model $y\left(t\right)=\mathop{\sum }\nolimits_{f=1}^{F}\mathop{\sum }\nolimits_{\tau =0}^{T}{{\mathbf{\upbeta }}}_{f}^{T}(\tau ){{\mathbf{x}}}_{f}\left(t-\tau \right)+\epsilon$ for each electrode, where *y* is the high-gamma activity recorded from the electrode, **x**_*f*_ (*t* − *τ*) is the stimulus representation vector of feature set *f* at time *t* *−* *τ*, **β**_*f*_(*τ*) is the regression weight for feature set *f* at time lag *τ*, and *ε* represents the Gaussian noise.

To prevent model overfitting, we used L2 regularization and cross-validation. Specifically, we divided the data into three mutually exclusive sets representing 80%, 10% and 10% of samples. The first set (80% of samples) was used as the training set. The second set was used to optimize the L2 regularization hyperparameter, and the final set was used as the test set. We evaluated the models using the correlation between the actual and predicted values of neural activity on held-out data. We performed this procedure five times, and the performance of the model was calculated as the mean performance across all testing sets.

The performance of each encoding model on an individual recording site (electrode/neuron) was quantified as the (normalized) BPS. In particular, BPS = ${R}_{{\textrm {model}}}^{2}/{R}_{{\textrm {baseline}}}^{2}$, where ${R}_{{\textrm {model}}}^{2}$ is the *R*^2^ value of the prediction model based on cross-validation and ${R}_{{\textrm {baseline}}}^{2}$ is the *R*^2^ value of the baseline model (full-feature set) for the same electrode/neuron based on cross-validation. A BPS of 1 indicates that the proposed model performs as well as the baseline model, and a BPS of >1 suggests that the proposed model outperforms the baseline model.

For the STRF model and the baseline full-feature model, we used a fixed delay-time window of 400 ms. For all DNN-based encoding models, we varied the time window length from 0 (using only the current timeframe) to 400 ms and selected the optimal window length based on cross-validation results.

### Noise-ceiling estimation

In one of the five TIMIT blocks (TIMIT5), ten sentences were repeated ten times. The noise ceiling in each electrode was computed using this repeat block. Let ${s}_{i,\,j}^{(k)}\in {{\mathbb{R}}}^{{T}_{i}}$ be the recorded signal in electrode *k* for the *j* th repetition of the *i*th sentence, where *i* = 1, …, 10; *j* = 1, …, 10; and *T*_*i*_ is the length of the *i*th sentence. We used a cross-validation strategy to estimate the noise ceiling. Specifically, we computed the averaged response from nine repetitions and correlated the averaged response to the left-out trial. The averaged Pearson correlation coefficient across all repetitions was used as the estimated noise ceiling for this electrode: ${r}^{\,(k)}=\frac{1}{10}\mathop{\sum }\nolimits_{i=1}^{10}\frac{1}{10}\mathop{\sum }\nolimits_{j=1}^{10}{\mathrm {corr}}\left\langle \frac{1}{9}\,{\sum }_{n\ne j}{s}_{i,n}^{\left(k\right)\,},{s}_{i,\;j}^{\left(k\right)}\right\rangle$, and the *R*^2^ value was the square of the Pearson correlation coefficient.

### Electrode selection

To select speech-responsive electrodes and avoid numerical instability of the BPS caused by dividing the very small *R*^2^ values of the baseline model, we included only speech-responsive electrodes in our analysis. The responsive threshold was set as ${R}_{{\textrm {baseline}}}^{2}$ > 0.05.

### DNNs: model architectures

We used five different DNN models: HuBERT^15^, Wav2Vec 2 unsupervised version^14^, Wav2Vec 2 ASR supervised version^14^, HuBERT supervised version and Deep Speech 2 (ref. ^13^).

The HuBERT and Wav2Vec 2 models share the same architecture, consisting of a convolutional waveform encoder and a transformer BERT encoder^71^. The network uses 16-kHz raw sound waveforms as the input. The convolution encoder consisted of seven 512-channel, one-dimensional convolution layers with strides of 5, 2, 2, 2, 2, 2, 2 and kernel widths of 10, 3, 3, 3, 3, 2, 2. The convolution encoder downsampled the input to a 512-dimensional feature sequence at a 20-ms framerate (50 Hz). The output of the convolution encoder, noted as ‘CNN out’, was projected to a 768-dimensional space through a linear layer, noted as ‘CNN proj’, and fed into the BERT encoder. The architecture of the transformer encoder is similar to that of the BERT base model^71^, which consists of 12 identical transformer-encoder blocks, with an embedding dimension of 768, intermediate feedforward layer dimension of 3,072 and 12 attention heads in each layer.

The Deep Speech 2 model consists of a convolutional spectrogram encoder and a recurrent encoder. This model uses the spectrogram of the raw audio signal as the input. The spectrogram was computed using a short-time Fourier transform with 161 frequency components from 0 to 8 kHz, time window size of 0.02 s and a stride size of 0.01 s. The convolution encoder consisted of two 32-channel, two-dimensional convolution layers, with corresponding two-dimensional strides of 2, 2 and 2, 1 and kernel sizes of 41, 11 and 21, 11. The final output of the convolution encoder was a 1,312-dimensional vector at a 20-ms framerate (50 Hz). The recurrent encoder consisted of five bidirectional LSTM layers, each with a hidden-state size of 1,024. The output of the last LSTM layer was projected to a 29-dimensional feature space by a linear projection layer.

### DNNs: unsupervised training

The HuBERT model was trained using a self-supervised paradigm of masked prediction^15^. The unsupervised *k*-means clustering algorithm was used to generate categorical labels of the acoustic speech signal, mimicking pseudophonetic labels. During training, a random subset of segments in each sentence was selected and masked. After masking, the sequence was passed through the network to generate a feature-embedding sequence. The embedded sequence was then projected to compute cross-entropy loss over discrete code categories.

The Wav2Vec 2 unsupervised model was trained using a self-supervised contrastive learning paradigm^14^. This model uses a quantization module to discretize the output sequence of the convolution encoder. Similar to the HuBERT model, a random subset of speech segments was selected and masked. The final output of the transformer encoder and the quantized representation from the convolution encoder were used to compute the contrastive loss. Specifically, for the target output at a given masked timestep, a random set of distractors was selected from other masked portions in the same sentence. The contrastive loss maximizes the distance between the target and the discretized output in the distractors while minimizing the distance between the target and the discretized output at the target timestep.

Both English models were trained on the 960-h LibriSpeech corpus^37^. For the cross-language comparison, we also trained a HuBERT Mandarin model on the 755-h MAGICDATA corpus of Mandarin speech^72^, using the same procedure as in the English HuBERT model and starting from random initializations.

We trained both the English and Mandarin self-supervised models for two iterations on 32 graphics processing units (GPUs), with a batch size of at most 87.5 s of audio per GPU. The first iteration was trained for 250,000 steps, whereas the second iteration was trained for 400,000 steps using labels generated by clustering the output of the sixth transformer layer in the first iteration. Training for 100,000 steps took ~9.5 h. The Adam optimizer was used with epsilon = 1 × 10^−6^, beta = (0.9, 0.98) and the learning rate ramped linearly from zero to the peak learning rate of 5 × 10^−4^ for the first 8% of the training steps and then decayed linearly back to zero.

Data augmentation was applied between the CNN and transformer modules. Temporal masks spanned ~200 ms, with a 0.08 probability of selecting a timestep as the beginning of a mask. We also masked channels by choosing several channels as starting indices and then covered the following 64 channels. Temporal and channel spans may overlap.

### DNNs: supervised training

The Wav2Vec 2 supervised model was fine-tuned from the unsupervised pretrained initialization^14^. A linear projection layer was used to project the output of the transformer encoder onto 29 classes representing characters, spaces and word boundaries. The model was optimized by minimizing a connectionist temporal classification (CTC) loss^73^. During fine-tuning, the weights of the convolution encoder were frozen and only the transformer layers were fine-tuned.

The HuBERT/Wav2Vec 2 supervised model was trained using a CTC loss. The entire weights of the CNN and transformer layers were trained altogether from random initializations.

The Deep Speech 2 model was trained, from random initializations, for the best ASR performance by minimizing the CTC loss^13^. The 960-h LibriSpeech corpus was used for the supervised training of all models.

### Attention pattern analysis

For a given speech sentence, assume that the embedding sequence in a transformer layer was of length *T* (*c*_1_, …, *c*_*T*_), the phoneme boundaries were indexed as *p*_1_, …, *p*_*m*_ and the syllable boundaries were indexed as *s*_1_, …, *s*_*n*_. The attention templates were defined as follows:

1. Attention to the current phoneme, phoneme(0): ${A}_{{\textrm {ph}}(0)}\in {{\mathbb{R}}}^{T\times T}$, ${A}_{{\textrm {ph}}(0)}\left(i,j\right)=1$ if ${p}_{k}\le i < {p}_{k+1}$ and ${p}_{k}\le j < {p}_{k+1}$ for any *k*; ${A}_{{\textrm {ph}}(0)}\left(i,j\right)=0$ otherwise.
2. Attention to the previous phoneme, phoneme(−1): ${A}_{{\textrm {ph}}(-1)}\in {{\mathbb{R}}}^{T\times T}$, ${A}_{{\textrm {ph}}(-1)}\left(i,j\right)=1$ if ${p}_{k}\le i < {p}_{k+1}$ and ${p}_{k-1}\le j < {p}_{k}$ for any *k*; ${A}_{{\textrm {ph}}(-1)}\left(i,j\right)=0$ otherwise.
3. Attention to the second to the previous phoneme, phoneme(−2): ${A}_{{\textrm {ph}}(-2)}\in {{\mathbb{R}}}^{T\times T}$, ${A}_{{\textrm {ph}}(-2)}\left(i,j\right)=1$ if ${p}_{k}\le i < {p}_{k+1}$ and ${p}_{k-2}\le j < {p}_{k-1}$ for any *k*; ${A}_{{\textrm {ph}}(-2)}\left(i,j\right)=0$ otherwise.
4. Attention to the current syllable, syllable(0): ${A}_{{\textrm {sy}}(0)}\in {{\mathbb{R}}}^{T\times T}$, ${A}_{{\textrm {sy}}(0)}\left(i,j\right)=1$ if ${s}_{k}\le i < {s}_{k+1}$ and ${s}_{k}\le j < {s}_{k+1}$ for any *s*; ${A}_{{\textrm {ph}}(0)}\left(i,j\right)=0$ otherwise. To exclude the current phoneme from the current syllable, we used ${A}_{{\textrm {sy}}(0)}^{{\prime} }={A}_{{\textrm {sy}}(0)}-{A}_{{\textrm {ph}}(0)}$ as the template.
5. Attention to the previous syllable, syllable(−1): ${A}_{{\textrm {sy}}(-1)}\in {{\mathbb{R}}}^{T\times T}$, ${A}_{{\textrm {sy}}(-1)}\left(i,j\right)=1$ if ${s}_{k}\le i < {s}_{k+1}$ and ${s}_{k-1}\le j < {s}_{k}$ for any *k*; ${A}_{{\textrm {sy}}(-1)}\left(i,j\right)=0$ otherwise.
6. Attention to the second to the previous syllable, syllable(−2): ${A}_{{\textrm {sy}}(-2)}\in {{\mathbb{R}}}^{T\times T}$, ${A}_{{\textrm {sy}}(-2)}\left(i,j\right)=1$ if ${s}_{k}\le i < {s}_{k+1}$ and ${s}_{k-2}\le j < {s}_{k-1}$ for any *k*; ${A}_{{\textrm {sy}}(-2)}\left(i,j\right)=0$ otherwise.

For each sentence, we computed the attention matrix *W*_*xy*_ at the *x*th layer and *y*th attention head. The correlation coefficient corr (*W*_*xy*_, *A*_*q*_) was computed for all templates. Moreover, the AS for layer *x* and template *q* was computed as the average over all attention heads and all speech sentences.

### STG clustering analysis

To identify functional clusters in the STG, we used a similar clustering approach as described previously^38^. Note that, instead of using raw single-trial responses, we averaged across sentences and used only averaged time series. Specifically, we applied convex non-negative matrix factorization (convex NMF)^74^ to decompose the averaged high-gamma time series across all STG electrodes. Specifically, $X\approx \hat{X}=F{G}^{T}$ and *F* = *XW*, where *X* (*T* time points × *p* electrodes) is the ERP matrix for different STG electrodes averaged across all sentences, *G* (*p* electrodes × *k* clusters) represents the spatial weight of each electrode for each cluster and *W* (*p* electrodes × *k* clusters) represents weights applied to the electrode time series. In particular, for *X*, we considered all 144 speech-responsive STG electrodes across all nine participants and computed the averaged ERP response for each electrode across all 599 TIMIT sentences. We evaluated different *k* values ranging from 1 to 10 and computed the percentage of variance explained by NMF models with different *k* values. We chose the number of clusters at the elbow of the variance curve (Extended Data Fig. 6), which yielded *k* = 2, and explained 94% of the total variance.

After choosing the optimal number of clusters, each electrode was assigned to a cluster with the maximum cluster weight *G*.

### Statistical testing

We used paired *t* tests (one-sample) to evaluate and compare the performance of DNN-based encoding models and the baseline models. In particular, the performance of different models was evaluated and compared on individual electrodes/units in each area. The d.f. of the *t* statistic was determined by the total number of individual electrodes/units in each area. Two-tailed *P* values were used to determine statistical significance. We also evaluated the effects in single-participant results (Extended Data Figs. 9 and 10). Data distribution was assumed to be normal, but this was not formally tested.

We used permutation tests to evaluate the statistical significance of the cross-layer correlations between BPSs and ASs in each DNN-layer prediction model for Figs. 4–6. In particular, we randomly shuffled speech sentences 800 times to disrupt the speech-neural correspondence, and reran the corresponding encoding models to compute *R*^2^ and obtain the surrogated distribution of the correlation coefficients. One-sided *P* values were estimated using this empirical distribution of correlation coefficients.

### Reporting summary

Further information on research design is available in the Nature Portfolio Reporting Summary linked to this article.

## Online content

Any methods, additional references, Nature Portfolio reporting summaries, source data, extended data, supplementary information, acknowledgements, peer review information; details of author contributions and competing interests; and statements of data and code availability are available at 10.1038/s41593-023-01468-4.

### Supplementary information

Reporting Summary

Supplementary Data 1 Editorial assessment report.

### Source data

Source Data Figs. 2–6 and Extended Data Figs. 3–10 Statistical source data.

## Data Availability

The LibriSpeech dataset is available at https://www.openslr.org/12. The MAGICDATA dataset is available at https://www.openslr.org/68/. The TIMIT dataset is available at 10.35111/17gk-bn40. The ASCCD dataset is available at http://paslab.phonetics.org.cn/?p=1763. Deidentified patient data that support the findings of this study will be made available from the corresponding author upon request. Source data are provided with this paper.

## Code availability

The completely developed code that operates on the full dataset will be made available from the authors upon reasonable request. Source code that implements the core neural encoding algorithm and the DNN analysis can be found at https://github.com/yuanningli/neural_encoding_demo.
