# Supplementary material for: Dissecting neural computations in the human auditory pathway using deep neural networks for speech
Source: Nat Neurosci. 2023 Oct 30;26(12):2213–25. doi: 10.1038/s41593-023-01468-4 (PMC10689246; doi:10.1038/s41593-023-01468-4)
Supplement: Supplementary file 1 — Reporting Summary [file 41593_2023_1468_MOESM1_ESM.pdf]

nature portfolio

Corresponding author(s): Edward Chang
Last updated by author(s): Aug 23, 2023

Reporting Summary

Nature Portfolio wishes to improve the reproducibility of the work that we publish. This form provides structure for consistency and transparency in reporting. For further information on Nature Portfolio policies, see our Editorial Policies and the Editorial Policy Checklist.

Statistics

For all statistical analyses, confirm that the following items are present in the figure legend, table legend, main text, or Methods section.

n/a	Confirmed	
☐	☒	The exact sample size (n) for each experimental group/condition, given as a discrete number and unit of measurement
☐	☒	A statement on whether measurements were taken from distinct samples or whether the same sample was measured repeatedly
☐	☒	The statistical test(s) used AND whether they are one- or two-sided<br>*Only common tests should be described solely by name; describe more complex techniques in the Methods section.*
☐	☒	A description of all covariates tested
☐	☒	A description of any assumptions or corrections, such as tests of normality and adjustment for multiple comparisons
☐	☒	A full description of the statistical parameters including central tendency (e.g. means) or other basic estimates (e.g. regression coefficient) AND variation (e.g. standard deviation) or associated estimates of uncertainty (e.g. confidence intervals)
☐	☒	For null hypothesis testing, the test statistic (e.g. F, t, r) with confidence intervals, effect sizes, degrees of freedom and P value noted<br>*Give P values as exact values whenever suitable.*
☒	☐	For Bayesian analysis, information on the choice of priors and Markov chain Monte Carlo settings
☒	☐	For hierarchical and complex designs, identification of the appropriate level for tests and full reporting of outcomes
☐	☒	Estimates of effect sizes (e.g. Cohen's d, Pearson's r), indicating how they were calculated

Our web collection on statistics for biologists contains articles on many of the points above.

Software and code

Policy information about availability of computer code

Data collection	Data collection used MATLAB R2014a, TDT Synapse v96
Data analysis	Data analysis used freesurfer 7.2, python 3.6, pytorch 1.10, fairseq 0.10, huggingface transformers 4.18, numpy 1.20, scipy 1.7, pandas 1.3, librosa 0.9, and scikit-learn 0.20.<br>The completely developed code that operates on the full data set will be made available from the authors upon reasonable request. A sample code that implements the core neural encoding algorithm and the DNN analysis can be found at https://github.com/yuanningli/neural_encoding_demo.

For manuscripts utilizing custom algorithms or software that are central to the research but not yet described in published literature, software must be made available to editors and reviewers. We strongly encourage code deposition in a community repository (e.g. GitHub). See the Nature Portfolio guidelines for submitting code & software for further information.

Data

Policy information about availability of data

All manuscripts must include a data availability statement. This statement should provide the following information, where applicable:

- Accession codes, unique identifiers, or web links for publicly available datasets
- A description of any restrictions on data availability
- For clinical datasets or third party data, please ensure that the statement adheres to our policy

Librispeech dataset is available at https://www.openslr.org/12. MAGICDATA dataset is available at https://www.openslr.org/68/. TIMIT dataset is available at https://doi.org/10.35111/17gk-bn40. ASCCD dataset is available at http://paslab.phonetics.org.cn/?p=1763. The de-identified patient data that support the findings of this study will be made available from the corresponding author upon request.

Field-specific reporting

Please select the one below that is the best fit for your research. If you are not sure, read the appropriate sections before making your selection.

☒ Life sciences ☐ Behavioural & social sciences ☐ Ecological, evolutionary & environmental sciences

For a reference copy of the document with all sections, see nature.com/documents/nr-reporting-summary-flat.pdf

Life sciences study design

All studies must disclose on these points even when the disclosure is negative.

Sample size	No explicit sample size calculation was performed. The amount of data collected from each participant was purely dependent on their clinical treatment schedule and the amount of time each participant was willing to volunteer for the study.<br>The sample size (n = 12 subjects) is greater than previous studies from our lab that successfully use intracranial ECoG (e.g. Chang et al. Nature Neuroscience 2010, n = 4; Mesgarani et al. Science 2014, n =6; Tang et al. Science 2017, n = 10). The number of task-relevant speech responsive electrodes (n = 144 in total for superior temporal gyrus, n = 53 in total for heschl's gyrus) is also comparable to similar psychophysical studies using high-density, broad-coverage ECoG grids.
Data exclusions	No data were excluded from analysis.
Replication	No explicit attempt at replication of the results reported has been undertaken. All encoding results were estimated on separated test set using cross-validation.
Randomization	The sentences and paragraphs within the speech corpora were randomly ordered.
Blinding	Blinding was not relevant for this study. The participants' task was to passively listen to the speech, and the experimenter did not interact with the participant during the experiment blocks.

Reporting for specific materials, systems and methods

We require information from authors about some types of materials, experimental systems and methods used in many studies. Here, indicate whether each material, system or method listed is relevant to your study. If you are not sure if a list item applies to your research, read the appropriate section before selecting a response.

Materials & experimental systems

n/a	Involved in the study	
☒	☐	Antibodies
☒	☐	Eukaryotic cell lines
☒	☐	Palaeontology and archaeology
☒	☐	Animals and other organisms
☐	☒	Human research participants
☒	☐	Clinical data
☒	☐	Dual use research of concern

Methods

n/a	Involved in the study	
☒	☐	ChIP-seq
☒	☐	Flow cytometry
☒	☐	MRI-based neuroimaging

Human research participants

Policy information about studies involving human research participants

Population characteristics	This study included 12 participants (6 male, 6 female, age from 31 to 55, all right-handed) who were neurosurgical patients at either UCSF or Huashan Hospital. These include patients with intractable epilepsy who had high-density electrode grids implanted for clinical monitoring of seizure activity, and eloquent brain tumor patients undergoing awake language mapping as part of their surgery.
Recruitment	Only the patients undergoing awake surgery with direct cortical stimulation were asked to participant in the study. We only included those participants with tumors which did not obviously invade the auditory cortex. All patients have normal hearing and intact speech cognitions, therefore we do not expect selection bias from the population. The placements of the grids were determined solely by clinical needs. All patients were clearly informed (as detailed in the IRB-approved written consent document signed by the participant) that the participation in the scientific research was completely voluntary and would not directly impact their clinical care. Additional verbal consent was also acquired at the beginning and during the breaks of each experiment session. The participants were compensated $50 for each session of experiment.
Ethics oversight	The experimental protocol was approved by the Institutional Review Board at the University of California, San Francisco (UCSF) and by the Huashan Hospital Institutional Review Board of Fudan University. All participants gave their written, informed consent prior to testing.

Note that full information on the approval of the study protocol must also be provided in the manuscript.
