# Supplementary material for: Dissecting neural computations in the human auditory pathway using deep neural networks for speech
Source: Nat Neurosci. 2023 Oct 30;26(12):2213–25. doi: 10.1038/s41593-023-01468-4 (PMC10689246; doi:10.1038/s41593-023-01468-4)
Supplement: Supplementary file 2 — Editorial assessment report. [file 41593_2023_1468_MOESM2_ESM.pdf]

Contents of this report

1. **Manuscript details**: overview of your manuscript and the editorial team.
2. **Review synthesis**: summary of the reviewer reports provided by the editors.
3. **Editorial recommendation**: personalized evaluation and recommendation from all 3 journals.
4. **Annotated reviewer comments**: the referee reports with comments from the editors.
5. **Open research evaluation**: advice for adhering to best reproducibility practices.

About the editorial process

Because you selected the **Nature Portfolio Guided Open Access** option, your manuscript was assessed for suitability in three of our titles publishing high-quality work in your field of research. More information about Guided Open Access can be found here.

Collaborative editorial assessment

Your editorial team discussed the manuscript to determine its suitability for the Nature Portfolio Guided OA pilot. Our assessment of your manuscript takes into account several factors, including whether the work meets the technical standard of the Nature Portfolio and whether the findings are of immediate significance to the readership of at least one of the participating journals in the Guided OA pilot.

Peer review

Experts were asked to evaluate the following aspects of your manuscript:

- **Novelty** in comparison to prior publications;
- **Likely audience** of researchers in terms of broad fields of study and size;
- **Potential impact** of the study on the immediate or wider research field;
- **Evidence** for the claims and whether additional experiments or analyses could feasibly strengthen the evidence;
- **Methodological detail** and whether the manuscript is reproducible as written;
- Appropriateness of the **literature review**.

Editorial evaluation of reviews

Your editorial team discussed the potential suitability of your manuscript for each of the participating journals. They then discussed the revisions necessary in order for the work to be published, keeping each journal's specific editorial criteria in mind.

Journals in the Nature portfolio will support authors wishing to transfer their reviews and (where reviewers agree) the reviewers' identities to journals outside of Springer Nature.
If you have any questions about review portability, please contact our editorial office at guidedoa@nature.com.

Manuscript details

Tracking number	Submission date	Decision date	Peer review type
GUIDEDOA-22-00463	**Apr 14, 2022**	**Aug 19, 2022**	Single-blind

Manuscript title	Author details
Dissecting neural computations of the human auditory pathway using deep neural networks for speech<br><br>**Preprint:** link if available	Edward Chang<br><br>**Affiliation:** University of California, San Francisco

Editorial assessment team

**Primary editor**	**Sachin Ranade**<br>Home journal: ***Nature Neuroscience***<br>ORCID: **0000-0002-5150-5776**<br>Email: **sachin.ranade@us.nature.com**
**Other editors consulted**	**David Rowland**<br>**Home journal: Nature**<br><br>**Cody Walters**<br>**Nature communications**<br>**ORCID:**
**About your primary editor**	Sachin received his PhD from Stony Brook University for his work on the responses of serotonin neurons in the dorsal raphe nucleus of rats engaged in an olfactory perceptual decision task. During his postdoctoral research at Cold Spring Harbor Laboratory he developed optogenetic tagging techniques to identify neural activity from distinct neuronal subtypes in mice to investigate their behavioral correlates. Prior to joining

	Nature Neuroscience, he was an editor at Nature Communications. His research interests include neural mechanisms of behavioral function and dysfunction at the circuit, systems, and computational level. Sachin is based in the New York office.

Editorial assessment and review synthesis

**Editor's summary and assessment**	The authors compare various deep network model performance in predicting responses to speech from intracranial recordings in the auditory pathway.
**Editorial synthesis of reviewer reports**	The two reviewers have combined expertise in DNNs, speech and language coding, and intracranial electrophysiology. Both reviewers find the approach to be significant and the questions to be important, but the manuscript is dense and have suggested additional analyses, clarification in some instances, better statistical reporting, model comparisons etc.

Editorial recommendation

Nature<br><br>Revision not invited	
Nature Neuroscience<br><br>Major revisions	
Nature Communications<br><br>Major revisions	

Next steps

**Editorial recommendation 1:**	Our top recommendation is to revise and resubmit your manuscript to *Nature Neuroscience.*
**Editorial recommendation 2:**	You may also choose to revise and resubmit your manuscript to *Nature Communications.*
**Note**	As stated on the previous page *Nature* is not inviting a revision at this time. Please keep in mind that the journal will not be able to consider any appeals of their decision through Guided Open Access.

Revision

To follow our recommendation, please upload the revised manuscript files using **the link provided in the decision letter**.

Revision checklist

- ☐ Cover letter, stating to which journal you are submitting
- ☐ Revised manuscript
- ☐ Point-by-point response to reviews
- ☐ Updated Reporting Summary and Editorial Policy Checklist
- ☐ Supplementary materials (if applicable)

Submission elsewhere

If you choose not to follow our recommendations, you can still take the reviewer reports with you.

Option 1: Transfer to another Nature Portfolio journal
Springer Nature provides authors with the ability to transfer a manuscript within the Nature Portfolio, without the author having to upload the manuscript data again. To use this service, **please follow the transfer link provided in the decision letter.** If no link was provided, please contact guidedOA@nature.com.

> *Note that any decision to opt in to In Review at the original journal is not sent to the receiving journal on transfer. You can opt in to In Review at receiving journals that support this service by choosing to modify your manuscript on transfer.*

Option 2: Portable Peer Review option for submission to a journal outside of Nature Portfolio
If you choose to submit your revised manuscript to a journal at another publisher, we can share the reviews with another journal outside of the Nature Portfolio if requested. You will need to request that the receiving journal office contacts us at guidedOA@nature.com. We have included editorial guidance below in the reviewer reports and open research evaluation to aid in revising the manuscript for publication elsewhere.

Annotated reviewer reports

The editors have included some additional comments on specific points raised by the reviewers below, to clarify requirements for publication in the recommended journal(s). However, please note that all points should be addressed in a revision, even if an editor has not specifically commented on them.

Reviewer #1 information	
**Expertise**	Auditory neuroscience, DNN, intracranial electrophysiology
**Editor's comments**	

Reviewer #1 comments	
**Section**	**Annotated Reviewer Comments**
**Remarks to the Author: Overall significance**	The manuscript describes a study combining intracranial recordings to speech and pre-trained DNNs. The authors use three transformer-based models with a convolutional encoder, as well as a convolutional/recurrent model. The models are trained using a self-supervised loss (contrastive or masked prediction) or directly trained to predict characters using a CTC loss. The authors use regularized regression to predict intracranial responses from these features using regularized regression, and they compare performance with a baseline model, which includes a spectrogram, plus several additional hand-crafted speech features. They also measure the ability of their DNN model to predict other hand-crafted models of the auditory nerve and IC. The authors report a number of findings, which I describe in detail under "strengths of the claims".<br><br>In general, I think the questions being addressed in this paper are important and I am excited that the Chang lab is tackling them. At present, the paper strikes as broad and shallow: it addresses many interesting questions, but none of the associated analyses and results are fleshed out to the degree that one would expect for a high-impact publication.
**Remarks to the Author: Strength of the claims**	Hierarchy and prediction accuracy<br>They report that the trained model outperforms their baseline model in STG but HG. The STG finding is a replication of the Kell study, using intracranial recordings (which is significant) and a more constrained stimulus set (speech). The HG result contradicts Kell though the methods, stimuli, and models are quite different. The Kell study is highly relevant and is barely discussed. The Kell study should be explicitly mentioned in the introduction, briefly noting its findings and limitations (fMRI being a major one), and the authors should discuss potential explanations for discrepancies in the Discussion (i.e., differences in recording methods, stimuli, models, training regime, etc.). The paper would be stronger if it directly engaged with the prior literature by attempting to investigate the causes of the differences between studies.<br><br>Why does it appear that delays are the same for all units in the AN and IC and why is this delay 50 ms? Presumably, the integration time for many auditory nerve units should be on the scale of milliseconds and the delay should be related to the

CF of the unit, and presumably, the integration time for IC units should be much longer? Similarly, why is the average TRF in the auditory nerve around 100 ms and why isn't the IC TRF much longer than the auditory nerve TRF? These results make one question the measures of the delay and integration time that are being reported.

The authors report that in the STG, performance improves with layer for more sustained responses but is relatively flat with layer for more transient responses. They seem to interpret this finding as arguing against hierarchical processing, but this interpretation doesn't follow. Just because all the layers of the DNN have transient activity to some degree does not imply that the transient responses in the brain reflect a parallel stream of processing. In my opinion, the flat prediction scores for the transient response are hard to interpret. I think it's a nice analysis, but the kind of thing that would be more appropriate for a supplemental figure.

The authors should plot the actual r^2 values not the normalized values ("brain prediction score"). They can rescale the axes across regions and explain in the legend and text why the absolute r^2 values will vary across regions for uninteresting regions. Ideally, there would be some kind of ceiling r^2 value computed based on the reliability of the responses, but that may not be possible if there are no repetitions. The authors should also report the r^2 scores for a model with randomized weights (e.g., in supplemental).

Unsupervised performance
Overall, the authors find comparable performance between their supervised and unsupervised models, which I think is a potentially useful contribution. However, the authors do not report a purely supervised model using the same architecture, which would provide a useful comparison point. It would be preferable if the authors trained a couple of architectures on all the various losses described in the paper, and then compared their performance. There is also no information presented about the performance of the various models tested on a speech recognition task, and whether that performance is at all comparable to human speech recognition performance. This data is important for interpreting the findings of this study since many studies have found that performance is a key determinant of neural predictivity.

Context dependence
The authors report that deeper layers perform "context-dependent" computations that are aligned to phonemic and syllabic structures, and that the degree of alignment is related to neural prediction accuracy. The use of context is vague and ill-defined, and I don't find the evidence convincing. The authors report that the attention weights in the transformer are correlated with boundaries between speech segments and that this correlation increases as one moves up the network to some extent. However, the correlation is weak and there is no control model. For example, would one observe a similar trend if you simply shifted the matrices in Figure 4b by a few samples, such that the boundaries no longer aligned with meaningful segment durations? What if the boundaries between segments were drawn simply based on the acoustic envelope?

The authors report that their measure of context-dependence – the increase in correlation across layers between attention weights and their structural measures – is correlated with neural predictivity in the STG but not in other regions. This observation does not provide strong evidence that the STG is performing context-dependent computations. Lots of things change across the layers of DNN and any property that increases across layers will tend to be correlated with neural predictivity in STG and negatively correlated with neural predictivity in AN/IC.

The notion that an attention-based architecture uses context while a CNN does not is far too simplistic (and again it's not clear what is meant by context). Moreover, the CNN encoder they use is not comparable to the types of CNN architectures that are typically used for speech recognition, since it just serves to replace the spectrogram encoder, going from a very high-res, waveform input into a 50-Hz sampled representation. Feedforward CNNs are capable of learning highly complex nonlinear inputs that are highly sensitive to "context" (e.g., effects of coarticulation). If the authors want to make claims about CNNs vs. attention-based architectures they should use a modern CNN architecture with similar performance.

Language dependence
The authors report that an English-trained model gives better predictions for English vs. Mandarin speech in the STG of English speakers and that a Mandarin-trained model gives better predictions for Mandarin vs. English speech in the STG of Mandarin speakers, even though STRF prediction accuracy is comparable. I think this is potentially quite interesting but one is left with several additional questions. First, it would be helpful to have a direct comparison of the performance of English and Mandarin models for the same stimuli. Presumably, one would expect the English model to outperform the Mandarin model for English speech in English speakers, and vice-versa in Mandarin speakers. Perhaps the best thing would be to have a supplemental figure that showed the full 2 (native language) x 2 (stimulus language) x 2 (model language) so that the reader can decide for themselves whether the underlying prediction scores support the hypothesis. Second, it would be useful to know if this effect is also observed in HG or whether it is specific to the STG. Third, there is virtually no discussion of what is currently known about language-specific effects in the auditory cortex and how their work builds on this prior work, making novelty difficult to assess.

Other comments
The authors should describe the architectures and tasks in more detail in the Methods. The training recipe should also be described in more detail including any data augmentation techniques.

The anatomical organization in Figure 3a does not seem to replicate the author's prior findings showing a clear posterior vs. anterior distinction. This point should be discussed.

It should be made clearer throughout that the auditory nerve and IC predictions are based on a model of the auditory nerve and IC, not based on neural responses. This distinction should be clarified in the abstract and in Figure 2, i.e., the titles of the figures should be something like AN model, IC model, HG intracranial, STG intracranial.

It should be made clear whether the models are causal. If they are not, it seems this greatly complicates any analysis about integration time or delay. If the models are not causal, this limitation and its associated consequence should be explicitly noted and discussed.

The main text should give a brief description of all reported measures such as the delay time (i.e., the encoding window size that yielded the best prediction) so that the reader does not have to consult the methods before reading the main text.

Does Figure 2b plot delay times for the best performing layer?

	Figure 2d I believe is in many cases plotting different layers from the same model, which is confusing in my opinion. It would be better if the analyses of different layers and different models were clearly distinct.<br><br>The authors state: “HG on average had a consistent temporal integration window of 200 ms, while STG showed more diversified profiles, some with transient integration windows less than 200ms and some with significant sustained temporal integration longer than 400ms (Fig 2c).” This variation in STG is not evident in the figure making the claim hard to evaluate.<br><br>The authors state: “the periphery may play a more important computational role than primary auditory cortex”. This statement is based on the fact that their DNN model gives better predictions of AN/IC compared with HG. However, just because their model does not give good performance in HG does not imply that HG does not play an important computational role in speech processing. There are many other potential explanations, the most likely of which is that their DNN model is far from perfect, given the innumerable differences between their DNN model and the brain. The discussion of the poor prediction accuracy of HG is way overblown in my opinion and should be mostly removed, other than noting that there are many potential explanations for their model failure.<br><br>In the Discussion, the authors seem to claim that category selectivity is more prominent in visual cortex compared with auditory cortex. I don’t know of any study directly comparing the degree of category selectivity between auditory and visual cortex in any kind of comparable manner. This claim should be removed unless there is data to back it up.
**Remarks to the Author: Reproducibility**	Very little information is given on statistics. I’m guessing in Figure 2 they are reporting a t-test between pairs of models across electrodes with standard errors also across electrodes? If this is correct, then the variance across subjects is not taken into account. In general, all statistics should account for the major source of variation which in this study is likely to be subjects. If there are not enough patients to support across subject statistics, the authors could report within-subject statistics for all subjects (e.g., all subjects significant at $p < 0.05$). There should be a dedicated section in the methods describing the statistics.

Reviewer #2 information

**Expertise**	Speech and language coding, DNNs
**Editor’s comments**	

Reviewer #2 comments

**Section**	**Annotated Reviewer Comments**

Remarks to the Author	This manuscript titled "Dissecting neural computations of the human auditory pathway using deep neural networks for speech" uses multiple state-of-the-art deep-learning based speech recognition algorithms and compares the ability of classical models to predict neural response along the AN-IC-HG-STG pathway. Using these state-of-the-art models, the authors also track acoustic and phonetic information along this pathway.<br><br>I find this approach of comparing deep-learning models to brain activity fascinating and timely. I believe that such models, despite not being developed specifically as brain models, may nonetheless be very useful. I am also very impressed by the level of sophistication this work required.<br><br>Personally, I found the paper very technical and hard to follow, to the point where sometimes I didn’t understand the motivation for some of the analyses. I also have some specific concerns.<br><br>1. The BPS measure is the primary measure used, so I think it should be explained and motivated better. It appears that the most natural candidate is R*2 [and in fact, it is used in P.16 line 31]. Is the classic/spectrogram model always the Base-model for BPS? If so, I'd mention it at the beginning [if it is mentioned at the beginning, but I missed it - my apologies].<br><br>2. Referring to 1., on p.7 lines 1-10 the authors report the difference in variance explained (why not BPS), I would like to see the actual values (that their subtraction of gives the results reported).<br><br>3. Often the term "DNN-encoding models" is used, and I understand that it covers all four models described on page 6. However, I found it somewhat confusing that it was contrasted with "linear encoding models". Because a linear-regularized model (ridge) is always used to predict to the brain, I found it confusing. They both use linear models to predict the brain. Furthermore, I do not understand how the base model, which includes frequency-based features, is "linear". Please explain how "linear models" are linear.<br><br>4. It is unclear which of the 4 models the authors are referring to when they state results regarding the "transformer layer" [for example, p.7 lines 1-10].<br><br>5. The authors report some non-significant results throughout the paper. As an example, see p.7 line 3 ["3.4% more variance in HG at Transformer layer 1 ($t(53) = 1.20$, $p = 0.23$, two-sided)". A

non-significant difference cannot be used to make a conclusion. Even more problematic are lines 39 and 41 on page 13. Based on non-significant (or marginally significant) results, the authors conclude that lower-level acoustic representation is 41 largely shared across languages.

6. I think it will be easier to appreciate figure 2d if the units were correlation or R^2.

7. I do not understand why the delayed response can indicate increased TRW. Is it necessary that the area integrated the information over time (p.7 lines 12-23).

8. P.9 does not explain why clustering analysis is done and some details as to how. I would start with an explanation of why clustering analysis is done and some details as to how.

9. On page 9, the authors provide a fascinating analysis that involves extracting the attention heads' weights (it is not clear which model of the four transformer-based models is used in this analysis). However, I am still not sure what this analysis means. Why is the author looking at these weights? Do they contain different information?

10. Some information about the fine-tuning of HuBert to Mandarin should be given (which/how-many layers were fine-tuned, dataset size etc.).

As I noted earlier I find the idea interesting and the analysis sophisticated but not always well-motivated or explained. I hope that my comments will help the authors to better convey their findings.

Open research evaluation

General information

Guidelines for Transparency and Openness Promotion (TOP) in Journal Policies and Practices ("TOP Guidelines")

The recommendations and requests in the table below are aimed at bringing your manuscript in line with common community standards as exemplified by the TOP Guidelines. While every publisher and journal will implement these guidelines differently, the recommendations below are all consistent with the policies at Nature Portfolio. In most cases, these will align with TOP Guidelines Level 2.

FAIR Principles

The goal of the recommendations in the table below related to **data or code** availability is to promote the FAIR Guiding Principles for scientific data management and stewardship (*Scientific Data* **3**: 160018, 2016). The FAIR Principles are a set of guidelines for improving 4 important aspects of digital research objects: **F**indability, **A**ccessibility, **I**nteroperability and **R**eusability.

ORCID

ORCID is a non-profit organization that provides researchers with a unique digital identifier. These identifiers can be used by editors, funding agencies, publishers, and institutions to reliably identify individuals in the same way that ISBNs and DOIs identify books and articles. Thus the risk of confusing your identity with another researcher with the same name is eliminated. **The ORCID website** provides researchers with a page where your comprehensive research activity can be stored.

Springer Nature collaborates with the ORCID organization to ensure that your research contributions (as authors and peer reviewers) are correctly attributed to you. Learn more at https://www.springernature.com/gp/researchers/orcid

Data availability
**Data Availability Statement**

Many journals, including all Nature Portfolio journals, require a Data Availability Statement in the manuscript as a condition of publication. The Data Availability Statement should be as detailed as possible and include accession codes or other unique IDs for deposited data, information about where source data can be found, and specify any restrictions to data access that may apply. At a minimum, the statement should indicate that data are available upon request and explain how data access can be granted. If data access is not possible, the reasons for this must be made clear in the Data Availability Statement.<br><br>More information about the Nature Portfolio data availability policy can be found here: https://www.nature.com/nature-portfolio/editorial-policies/reporting-standards#availability-of-data<br><br>Additional information about Data Availability Statements and Springer Nature's data policies are available here: http://www.springernature.com/gp/authors/research-data-policy/data-availability-statements/12330880
**Mandatory data deposition**
To promote transparency in research, your neuroimaging data should be deposited in a community-endorsed, public repository. Details for accessing the data should be provided in the Data Availability Statement.<br><br>We recommend the following repositories:<br><br>-OSF (https://osf.io/) for neuroimaging raw data and EEG/EMG/MEG raw data<br>-Neurovault (https://neurovault.org/) for unthresholded statistical maps, parcellations, and atlases produced by MRI and PET studies.<br><br>More information on mandatory data deposition policies at the Nature Portfolio can be found at http://www.nature.com/authors/policies/availability.html#data<br><br>Please visit https://www.springernature.com/gp/authors/research-data-policy/repositories/12327124 for a list of approved repositories for various data types.
**Other data requests**

In line with community standards regarding open research, Springer Nature strongly supports data sharing and believes that all datasets on which the conclusions of the paper rely should be available to readers. We encourage authors to ensure that their datasets are either deposited in publicly available repositories (where available and appropriate) or presented in the main manuscript or additional supporting files whenever possible.<br><br>To learn more about data sharing and recommended data repositories, please see https://www.springernature.com/gp/authors/research-data-policy/repositories/12327124
**Data publishing recommendations**
**Code availability and citation**
To adhere to community standards and promote transparency in research, any custom software or code should be made publicly available, ideally before publication so that referees can test the code and comment on it.<br><br>Please include a statement under the heading "Code Availability", indicating whether and how the custom code/software reported in your study can be accessed, including any restrictions to access. This section should also include information on the versions of any software used, if relevant, and any specific variables or parameters used to generate, test, or process the current dataset. Code availability statements should be provided as a separate section after the Data Availability section.<br><br>Upon publication, Nature Portfolio journals consider it best practice to release custom computer code in a way that allows readers to repeat the published results. Code should be deposited in a DOI-minting repository such as Zenodo, Gigantum or Code Ocean and cited in the reference list following the guidelines described in our policy pages (see link below). Authors are encouraged to manage subsequent code versions and to use a license approved by the open source initiative. Full details about how the code can be accessed and any restrictions must be described in the Code Availability statement.<br><br>See here for more information about Nature Portfolio's code availability policies: https://www.nature.com/nature-portfolio/editorial-policies/reporting-standards#availability-of-computer-code<br><br>We also provide a Code and Software submission checklist that you may find useful: https://www.nature.com/documents/nr-software-policy.pdf

Please note: because of advanced features used in this form, you must use Adobe Reader to open the document and complete it.

Ethics

We believe that research that involves the use of clinical, biomedical or biometric data from human participants must only be carried out with the explicit consent of those whose data are involved. Consent must be obtained without any form of coercion and with participants' explicit understanding of the purpose for which their data will be used.

Because your study includes human participants, confirmation that all relevant ethical regulations were followed is needed for publication in any Springer Nature journal, and that informed consent was obtained. This must be stated in the Methods section, including the name of the board and institution that approved the study protocol.

Further details about the Nature Portfolio policy can be found at https://www.nature.com/commsbio/editorial-policies/ethics-and-biosecurity

Statistical reporting

Wherever statistics have been derived (e.g. error bars, box plots, statistical significance) figure legends should provide and define the n number (i.e. the sample size used to derive statistics) as a precise value (not a range), using the wording "n=X biologically independent samples/animals/cells/independent experiments/n= X cells examined over Y independent experiments" etc. as applicable. The figure legends must also indicate the statistical test used. Where appropriate, please indicate in the figure legends whether the statistical tests were one-sided or two-sided and whether adjustments were made for multiple comparisons. For null hypothesis testing, please indicate the test statistic (e.g. F, t, r) with confidence intervals, effect sizes, degrees of freedom and P values noted.

All error bars need to be defined in the figure legends (e.g. SD, SEM) together with a measure of centre (e.g. mean, median). For example, the legends should state something along the lines of "Data are presented as mean values +/- SEM" as appropriate. All box plots need to be defined in the legends in terms of minima, maxima, centre, bounds of box and whiskers and percentile.

For examples of expected description of statistics in figure legends, please see the following: https://www.nature.com/articles/s41467-019-11636-5 or https://www.nature.com/articles/s41467-019-11510-4.

When describing results as "significant" in the main text, please include details about the statistical test used and provide an exact p-value, rather than a signifcance threshold.
**Data presentation**
Bar graphs should only be used to present counts or proportions. If you are using bar graphs that present means/averages, it is best practice to include individual data points and/or convert the graph to a boxplot or dot-plot. You may wish to refer to this blog post (https://ecrlife420999811.wordpress.com/2018/07/10/beyond-bar-graphs-free-tools-and-resources-for-creating-more-transparent-figures-for-small-datasets/) about representing data distribution in plots (particularly for small datasets).
